# Investigating human paleodiet at Mesolithic Yuzhniy Oleniy Ostrov, Karelia using a multi-proxy stable isotope approach

**DOI:** 10.1371/journal.pone.0338887

**Published:** 2026-01-07

**Authors:** Rebekka I. I. Eckelmann, Laura Arppe, Rick J. Schulting, Sambit Ghosh, Jakub Trubač, Aneta Kuchařová, Matthew J. Wooller, Dmitry Gerasimov, Vyacheslav Moiseyev, Kristiina Mannermaa

**Affiliations:** 1 Department of Cultures, University of Helsinki, Helsinki, Finland; 2 Finnish Museum of Natural History, University of Helsinki, Helsinki, Finland; 3 School of Archaeology, University of Oxford, Oxford, United Kingdom; 4 Alaska Stable Isotope Facility, Institute of Northern Engineering, University of Alaska Fairbanks, Fairbanks, Alaska, United States of America,; 5 Institute of Geochemistry, Mineralogy and Mineral Resources, Faculty of Science, Charles University, Prague, Czech Republic; 6 College of Fisheries and Ocean Sciences, University of Alaska Fairbanks, Fairbanks, Alaska, United States of America; 7 Peter the Great Museum of Anthropology and Ethnography (Kunstkamera), Russian Academy of Sciences, St Petersburg, Russia; Senckenberg Gesellschaft fur Naturforschung, GERMANY

## Abstract

This study employs multiple isotopic proxies to investigate dietary practices at Yuzhniy Oleniy Ostrov (YOO, Karelia, north-western Russia), the largest Mesolithic cemetery in northern Europe. Building on previous research, we combine bulk *δ*¹³C and *δ*¹⁵N collagen measurements from 60 human individuals with new *δ*¹³C values on enamel bioapatite (n = 10) and the first archaeological compound-specific isotope analysis of amino acids (CSIA-AA) from north-western Russia (n = 6), to provide a more comprehensive view of local resource use and dietary patterns. Results show that YOO forms a distinct isotopic cluster within the broader circum-Baltic hunter-gatherer-fisher spectrum, characterized by unusually high *δ*¹⁵N values for an inland site. CSIA-AA and combined enamel-collagen *δ*¹³C data do not indicate marine contributions or freshwater seal hunting as the drivers of these values, instead confirming a high reliance on freshwater resources compatible with the Lake Onega system and potentially an increased diet-to-tissue offset related to a high protein diet. FRUITS Bayesian dietary modelling similarly indicated a high freshwater component, alongside unexpectedly low contributions from terrestrial game and high estimates for plant foods. The latter results diverge from ethnographic expectations for boreal hunter-gatherer-fisher subsistence and may reflect current limitations in modelling lipid intake and adaptive strategies in high-protein diets, highlighting a need to reconsider how isotopic and ethnographic data are integrated. Isotopic variability between individuals was minimal, with no significant differences by sex or burial location, supporting a shared dietary regime during the relatively short period of site use. Overall, this study presents the most detailed isotopic evaluation of diet at Yuzhniy Oleniy Ostrov to date and underscores the value of multi-proxy isotope analysis in exploring prehistoric lifeways.

## Introduction

As one of the largest known Early Holocene burial sites with preserved skeletal remains in northern Europe, Yuzhniy Oleniy Ostrov (YOO, Karelia, north-western Russia) plays a key role in understanding subsistence and dietary practices in the northern reaches of human settlement in a time of change marked by the impact of the 8.2 ka climatic cold event. Due to its size and mortuary variability, including burials with hundreds of recovered grave goods as well as those without any, the site has long offered a unique opportunity to investigate early hunter-gatherer-fisher (HGF) lifeways and social organization. Dietary reconstructions provide one avenue through which archaeologists can approach these topics, as diet is closely linked to cultural and subsistence practices, as well as patterns of social interaction. One of the most important tools used for this purpose is stable isotope analysis.

At YOO, however, stable isotope analyses have so far been carried out primarily in the context of radiocarbon dating [1,2]. While these studies indicate a significant contribution of aquatic resources to the YOO diet and identify unusually high *δ* ^15^N values, they focused on assessing freshwater reservoir effects, rather than on detailed dietary reconstruction and its implications for subsistence and social differentiation. Here, we build on this foundation to investigate dietary intake in greater depth, integrating new and previously published bulk stable carbon and nitrogen isotope data from collagen (expressed as *δ*^13^C_col_ and *δ*^15^N_col_ respectively) and increasing the sample size from 41 to 60 individuals. We further conducted compound-specific stable carbon and nitrogen isotope analysis of amino acids (*δ*^13^C_AA_ and *δ*^15^N_AA_) and stable carbon isotope measurements on enamel bioapatite (*δ*^13^C_apa_) on a subsample of these individuals, making these the first analyses of this kind for archaeological materials from the Stone Age of north-western Russia (ca. 9000 BCE – 300 CE) [3–6].

By applying this multi-tissue, multi-proxy approach, we aim to refine dietary reconstruction at YOO, reassess the relative reliance on aquatic versus terrestrial resources, which may also aid in refining the radiocarbon reservoir corrections, and test long-standing hypotheses about freshwater seal hunting at Lake Onega and the broader subsistence economy in this region. In addition, we explore intra-site isotopic variability in relation to burial location and sex, which may reflect aspects of social organization or food sharing practices. Finally, we situate the YOO dataset within the wider isotopic framework of Late Mesolithic and Early Neolithic (distinguished in north-eastern Europe by the presence of pottery rather than of domesticates) HGF groups across northern Europe, to examine both regional distinctions and shared adaptive strategies in subsistence, mobility, and environmental resilience during the early Holocene.

### Stable isotopes in dietary reconstructions

Bulk *δ*^13^C and *δ*^15^N values measured on archaeological tissues have been used to investigate past dietary patterns since the late 1970s [3] and both methodological refinement and varied applications in archaeological research has been ongoing since then [4].

*δ*^13^C tracks the carbon isotopic signature of the dominant basal producer within the food web of a consumer and the primary carbon source they are using. This results in differences between systems based primarily on terrestrial plants using C_3_ or C_4_ photosynthetic pathways (ranging from −22‰ to −35‰ and −9‰ to −17‰ respectively) [3,5]. Differences also occur between these and marine (−12 to −17‰) and to a degree freshwater ecosystems, which are highly variable but often marked by relatively low *δ*^13^C values combined with relatively high *δ*^15^N values [6–8]. C_4_ plants are absent in the temperate boreal forest environment of northern Europe and are excluded from consideration in this study. Instead, the primary factors assumed to influence dietary *δ*^13^C values are the differences between aquatic and terrestrial resources. *δ*^13^C values are also influenced by a range of environmental factors affecting plant metabolism (e.g., heat stress, light availability, recycling of respired CO_2_ in closed forests) [9]. Additionally, there is a small trophic level increase in *δ*^13^C values of ca. 1‰ [10,11].

*δ*^15^N values are widely used as trophic level indicators, as they typically increase by approximately 3–6‰ with each step in a food chain [12–15]. This enrichment enables researchers to estimate the trophic position of a specimen and assess the amount and trophic level of protein in their diet [12,16,17]. In aquatic environments, where food chains are often longer, elevated *δ*^15^N values are commonly interpreted as evidence of high marine or freshwater protein consumption (HMP and HFP, respectively) [17,18]. However, variability at the base of the food web (e.g., differences in soil nitrogen availability, organic matter content, or aridity), or metabolic factors, can also impact *δ*^15^N values, complicating interpretations of trophic relationships and trophic level estimates based on *δ*^15^N_col_ measurements [18–20].

Beyond these differences in primary systems, archaeological dietary reconstructions are affected by the differences in isotopic composition between macronutrients (lipids, carbohydrates and protein) and their physiological routing during consumer tissue formation. The *δ*^15^N signal in omnivore collagen reflects protein intake, with no significant contribution of lipids and carbohydrates, which are usually summarized as “energy” [21]. Lipids have substantially lower *δ*^13^C values, ca. −8‰ compared to protein [21]. As a result of dietary routing the *δ*^13^C signal in collagen also mainly reflects the protein portion of the diet. This bias increases in high protein consumers [22,23]. In contrast, *δ*^13^C measured on enamel apatite represents overall diet without bias towards a specific macronutrient, which combined with the collagen proxies allows more comprehensive dietary assessments [24–27]. This potentially allows for a greater appreciation of the contribution of plant foods to the diet, which are underrepresented in the protein signal, and enables the differentiation of marine or C_4_ contributions to the different macronutrient units through a combination of *δ*^13^C_col_ and *δ*^13^C_apa_ values [26,28,29].

In archaeological research, Bayesian Mixing Models (BMMs) are frequently employed to estimate the proportional contributions of different food sources to the isotopic signature measured in a consumer [19]. However, the interpretation of stable isotope values and the effectiveness of BMMs depend on a robust understanding of the relevant local isotopic baseline and supporting background data (e.g., from relevant archaeological floral and faunal assemblages) to situate them. In cases like YOO, where local broad baseline sampling is not possible due to a paucity of suitable (i.e., unburnt) archaeological remains and the general scarcity of local isotope studies, it is prudent to compare alternative models, do sensitivity testing and evaluate model results critically. In addition, when baseline parameters are ambiguous, additional proxies like CSIA of individual amino acids for *δ*^13^C and more rarely *δ*^15^N are increasingly applied. This approach helps to provide more precise differentiation between food sources, metabolic, trophic and environmental processes, thereby improving the robustness of BMM estimates [30–35].

Amino acids are typically classified into essential amino acids (EAAs, e.g., leucine (Leu), isoleucine (Ile), phenylalanine (Phe), threonine (Thr) and valine (Val)), and non-essential amino acids (NEAAs, e.g., glutamic acid/glutamine (Glx) and glycine (Gly)). While NEAAs can be synthesized *de novo* by the consumer, often incorporating carbon from various metabolic pathways, EAAs must be obtained through the diet and are integrated into consumer tissues with minimal isotopic alteration. This classification is especially relevant in the context of *δ*^13^C_AA_ analysis, as the conservative behavior of *δ*¹³C values of essential amino acids (*δ*^13^C_EAA_) effectively preserves the *δ*^13^C_EAA_ signature of producers at the base of the food web (i.e., algae, fungi, bacteria and different plant types) [36,37] and forms the premise of the so-called ‘fingerprinting’ approach often applied in ecological studies [37,38]. Previous research indicates that the relative *δ*^13^C patterns among EAAs are largely conserved within basal producer groups, and by extension in their consumers. In theory, this means that the contribution of food deriving from different basal producers (e.g., algae, C_3_ plants, fungi and bacteria) can be tracked via the relationship of different *δ*^13^C_EAAs_ even without a detailed assessment of the local isotopic baseline. [41,42]

In archaeological studies, a more commonly employed approach is to use direct comparisons between EAAs of groups with known consumption patterns, e.g., using *Δ*^13^C_Lys-Phe_ and *Δ*^13^C_Gly-Phe_ as proxies to identify consumption of freshwater, marine and lagoonal resources [30,35,39,40].

For *δ*^15^N_AA_-based studies of trophic standing, amino acids are instead classified based on their isotopic behavior during trophic transfers, typically into trophic amino acids (AA_tr_, e.g., glutamic acid (Glx), proline (Pro), valine (Val)) which show substantial ^15^N-enrichment with increasing trophic level, and source amino acids (AA_src,_ e.g., lysine (Lys), phenylalanine (Phe)), which show very little change from the *δ*^15^N at the base of the food web [41,42]. This contrast is the basis for the use of *δ*^15^N_AAs_ as more precise indicators of trophic relationships than bulk nitrogen isotope values. Ecological studies often calculate trophic level, or position (TP), based on the difference in *δ*¹⁵N values between trophic and source amino acid values, most commonly between *δ*^15^N_Glx_ and *δ*^15^N_Phe_ [43–45]. However, recent studies have shown that the division into source and trophic amino acid does not remain clear-cut across species and age stages [41,42], and that TP estimates can also be influenced by variability in baseline *δ*¹⁵N values, isotopic offsets between trophic and source amino acids at the base of the food chain (*β* values), which may differ between ecosystems and basal producer groups [46], and trophic discrimination factors (TDFs), which can vary with physiology, diet quality, and metabolic routing [44,47,48].

Nonetheless, a key advantage of *δ*¹⁵N_AA_ analysis is that both source and trophic amino acids can be measured within the same sample, allowing internal calibration of trophic position without requiring extensive external baseline data, improving the resolution of dietary and ecological interpretations, particularly in archaeological contexts where baseline information is limited or absent [34,44,49].

### Archaeological and environmental background

**Fig 1:**
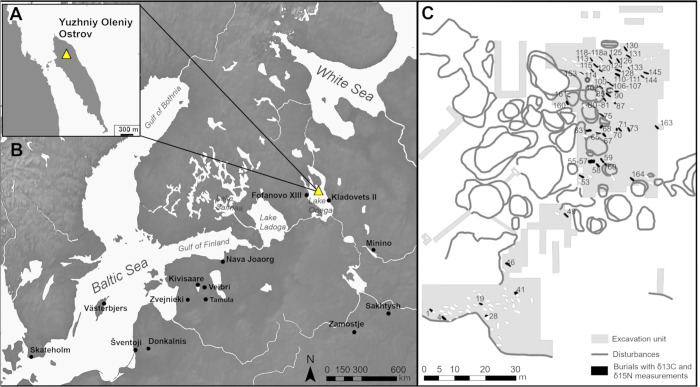
Location of Yuzhniy Oleniy Ostrov and northeast Baltic sites mentioned in the text with site detail. A) Detail of the location of Yuzhniy Oleniy Ostrov with (B) showing the site and others in a wider geographical context [51], with C) showing a map of the Yuzhniy Oleniy Ostrov site with sampled burials highlighted. The site map was created using Natural Earth public domain data [52].

Excavated in the 1930s, Yuzhniy Oleniy Ostrov (YOO) in Karelia, north-western Russia, is the only securely identified Stone Age burial site with skeletal preservation in a radius extending for two hundred kilometers (Fig 1) and the largest Mesolithic cemetery in northern Europe, encompassing a total of 177 identified burials (with many others having been destroyed during quarrying) and more than 7000 objects included in the graves, predominantly animal tooth ornaments [50]. Recent radiocarbon analyses date the burial site not only to the end phase of the local Mesolithic – defined as non-ceramic using HGF cultures in contrast to the Neolithic ceramic using HGFs – but also to a narrow time frame of ca. 6200–6000 cal BCE coinciding with the 8.2 ka cold event [1]. YOO is thus currently not just the only viable opportunity to apply modern bioarchaeological methods on human skeletal remains to the investigation of early Holocene HGF diet and subsistence in the area but also has the potential to reveal information on human responses to adverse environmental conditions. However, this line of inquiry is currently limited by the absence of local human samples dating to before or after the 8.2 ka cold event.

The site itself is located on a small eponymous island in the northern part of Lake Onega (62°07′44.3″N and 35°34′33.9″E, [53]), Europe’s second largest lake. With a modern surface area of ca. 9900 km^2^ it is the dominating feature of the local landscape and environment to the point where it creates its own microclimate [1,54,55]. The island’s surroundings are marked by high diversity in landscape features and ecozones, as well as some of the historically most productive fishing grounds in European Russia [56,57]. Considering the locality and ethnographic parallels with other Boreal hunter-gatherer communities, the assumption that aquatic resources would have been part of the subsistence of the YOO population has been present ever since the first publication on the site [52]. However, the extent of reliance on freshwater resources has remained unclear, and sometimes controversially discussed, due to the paucity of material evidence for fishing at the site compared to an abundance of finds associated with the hunting of terrestrial animals and their physical remains in the form of tooth pendants [52,58].

The first bioarchaeological analysis that included dietary investigations at YOO were based on trace element concentrations of strontium and calcium [59] and zinc and copper [60]. Both studies inferred differences between groups of individuals, in regard to meat intake [59], and the consumption of both protein and marine invertebrates [60]. Contemporary literature does not support the use of trace elements on archaeological bone for direct dietary inferences due to issues with diagenesis (for a review see [61]) and these studies are therefore not considered further here. In 2013 *δ*^13^C_col_ and *δ*^15^N_col_ values from four YOO individuals were published to contextualize Minino, another Mesolithic burial site, by Wood and colleagues [2] and the results showed high *δ*^15^N values indicating aquatic resource consumption [2]. In 2022, a larger set of *δ*^13^C_col_ and *δ*^15^N_col_ values with 38 sampled individuals was published by Schulting and colleagues [1] associated with a major radiocarbon dating effort at the site. They confirmed the widespread prevalence of a high aquatic diet component and included a small sample of faunal *δ*^13^C_col_ and *δ*^15^N_col_ values as baseline proxies which are also referenced here.

No evidence for diachronic trends or sex-based differences in dietary intake at the site has been identified in previous work [1], but unusually high *δ*^15^N_col_ values were noted [1,2], recalling previous hypotheses regarding the potential former presence of freshwater seals and seal hunting in Lake Onega [62,63]. Schulting et al. also pointed out high variability in freshwater reservoir effects that were interpreted as indicating that fish from different carbon reservoirs were consumed by the YOO community. This suggests that local baseline values may not have been homogenous even within the lake basin, connected to the still poorly understood isoscape of Lake Onega and its environs. Considering the lake’s size, depth differences, numerous straits, bays, smaller lakes and hundreds of tributaries, it is very likely substantial internal variation in isotopic compositions exists within the lake system [64,65].

Due to their emphasis on radiocarbon dating, both studies remained focused on the identification of freshwater resource consumption without a more detailed paleodietary reconstruction or integration of the socio-cultural context of the buried individuals. Here, we apply a multiproxy and multi-tissue strategy combining these previous results with new *δ*^13^C_col_ and *δ*^15^N_col_ values, additional *δ*^13^C_AA_ and *δ*^15^N_AA_ and *δ*^13^C_apa_ measurements and the application of Bayesian mixing models to provide more detailed characterization of past diet at YOO.

## Material

The archaeological samples analyzed in this study all originate from the Yuzhniy Oleniy Ostrov burial site. They are analyzed with the permission of the Anthropology and Archaeology sections of the Peter the Great Museum of Anthropology and Ethnography (MAE) in St. Petersburg, where they are curated in collections 5773 and 5716 (for details including specimen numbers see SI3, Table S1 in S1 Data). No further permits were required for the described study, which complied with all relevant regulations.

In previous studies [1,2], 41 humans were sampled to analyze for *δ*^13^C_col_ and *δ*^15^N_col_ [1,2] with all samples originating from collagen of adult bone and one third molar. In addition, 13 pendants made from Eurasian elk (*Alces alces*) incisors, one Eurasian beaver (*Castor fiber*) tooth pendant and dates for two bird bones – an osprey (*Pandion haliaetus*) and a black-throated diver (*Gavia arctica*) – were also available [1]. A wild boar (*Sus scrofa*) tooth was analyzed (ibid.) but excluded as a baseline value due to its likely non-local origin [66]. Bone collagen of two modern perch (*Perca fluviatilis*) and one vendace (*Coregonus albula*) from Lake Onega were also measured [1].

The new human measurements added in the present study were conducted exclusively on dental tissues (see SI3, Table S1 in S1 Data). *δ*^13^C_col_ and *δ*^15^N_col_ values from 19 additional individuals were obtained through sequential sampling of tooth dentine in 2021 (Eckelmann et al., in prep.), following the procedure described in [67], which employs a biopsy punch for sampling. The values included in this study represent an average of the microsamples formed from five years of age onwards [68] for each analyzed tooth, with age assignments following [67,68]. The cut-off age at five years was chosen to avoid isotopic signals affected by nursing effects [73,74], as the cessation of breastfeeding was identified in the serial profiles as occurring before four years of age in all individuals (Eckelmann et al., in prep., following [67,69]), and to ensure the values reflect diet based on environmental resources. This inclusion of both bone and tooth collagen samples means that the isotopic data represents different time scales and periods in the individuals’ lives, as primary dentine forms during childhood whereas bone values represent an isotopic signal averaged over multiple years before death [70,71]. Six of these newly sampled individuals were also analyzed for *δ*^13^C_AA_ and *δ*^15^N_AA_ on dentine collagen and ten for *δ*^13^C_apa_ of tooth enamel. Biological sex was determined via biomolecular analysis as part of a forthcoming PhD-dissertation (Batanina et al. in prep.) for all but five individuals for whom osteological evaluations were used (in brackets in Table 1). Their placement in spatial clusters (North (burials 1–47) and South (burials 48–170)) within the site was accepted as established in the literature [52,59,72]. An overview of the complete numbers of available human samples for each analysis is provided in Table 1.

**Table 1:** Summary of the archaeological human data currently available for each type of analysis. The numbers in brackets refer to sex identification based on osteology only (for details see SI3, Table S1-2 in S1 Data).

Analysis Material	*δ*^13^C_col_ & *δ*^15^N_col_ bone collagen	*δ*^13^C_col_ & *δ*^15^N_col_ dentine collagen	*δ*^13^C_AA_ *δ*^15^N_AA_ dentine collagen	*δ*^13^C_apa_ enamel apatite
Sampled Individuals	40	37	6	10
	in total: 60 individuals; 22(+2) females, 24 (+3) males; 9 unidentified		3 female, 3 male	4 female, 6 male
Source	[1,2]	[1], this paper	this paper	this paper

In addition to these human skeletal materials, the biological baseline was also expanded through further *δ*^13^C_col_ and *δ*^15^N_col_ measurements on the dentine collagen of two archaeological reindeer (*Rangifer tarandus*) molars (one first molar from burial 61 and one third molar from burial 138) and on the bone collagen of two additional modern perch and one modern zander (*Sander lucioperca*) from Lake Onega, the latter of which was also subjected to CSIA. Bone collagen of the vendace and of one perch previously sampled for bulk collagen isotopic analyses [1] was also re-sampled for CSIA (SI3, Table S2 in S1 Data). Accordingly, there are in total currently 16 herbivore, two bird and five fish *δ*^13^C_col_ and *δ*^15^N_col_ measurements from the Lake Onega area available for this analysis, in addition to three fish CSIA values.

## Laboratory analyses

Full descriptions of laboratory sample pre-treatment and isotopic measurements for all analyses are detailed in the Supporting Information SI1 in S1 File with a summary found in the following paragraphs. ^15^N/^14^N and ^13^C/^12^C ratios are presented in the *δ* notation with units of per mil (‰), relative to the international standards atmospheric N_2_ (AIR N_2_) and Vienna Peedee Belemnite (VPDB), respectively.

The new human and animal bulk collagen *δ*^13^C_col_ and *δ*^15^N_col_ samples were processed following the protocol of Czermak and colleagues [67], with the values given representing the post-weaning average of the sequence. The reindeer and fish samples similarly followed the demineralization protocol described in Czermak et al. [67]. Isotope data were normalized to USGS-40 (*δ*¹³C = −26.4‰ and *δ*¹⁵N = −4.5‰) and USGS-41 (*δ*¹⁵N = 47.6‰ and *δ*¹³C = 37.6‰). Quality control reference materials (caffeine, collagen) not used for normalization indicate a precision of ±0.2‰ for *δ*^13^C_col_ and ±0.2‰ for *δ*^15^N_col_ measurements (see SI1, Table S1 in S1 File).

For the analysis of *δ*^13^C_apa_, samples were prepared following pre-treatment after [73,74] aimed at retaining only the structural carbonate. The applied analytical procedure was based on Revesz et al. [75] with modifications. Calibration of the results versus the V-PDB scale was achieved using international reference materials NBS-18 (*δ*^13^C = −5.0‰) and IAEA-603 (*δ*^13^C = 2.5‰) (International Atomic Energy Agency, Vienna, Austria). The internal precision (SD) measured over eight replicates of the calcite materials used for calibration was 0.02‰ for raw *δ*^13^C values. The values presented are an average of multiple samples, typically seven (mean 7 ± 1), taken across the vertical axis of the tooth crown spanning from apex to enamel root junction and represent a mean of *δ*^13^C_apa_ incorporated during the complete time of enamel formation (for the sampled teeth this usually means from birth to ca. four years of age).

The CSIA-AA analyses and sample preparation were conducted following previously published protocols [49,76,77] and included six samples of human and three samples of modern lake Onega fish collagen remaining from previous bulk isotopic analyses. External UAF AA1 and AA2 standards were used to calibrate measurements. Accuracy was assessed by comparing measured and known values of an internal norleucine standard for both *δ*^13^C_AA_ (*Δ* = 0.1‰) and *δ*¹⁵N_AA_ (*Δ* = 1.1‰) and an external caffeine standard for *δ*¹⁵N_AA_ (*Δ* = 0.4‰) only. Data quality was monitored through the relationship between measured proline and hydroxyproline isotope values, both showing high correlation and regression lines close to the expected 1:1 line (*δ*^15^N_Hyp vs Pro_: R2 = 0.998, slope = 1.012 and *δ*^13^C_Hyp vs Pro_: R2 = 0.996, slope = 1.014), indicating good data quality (SI2, Fig S2 in S1 File) [78].

## Data processing

To all *δ*^13^C values derived from modern samples, a correction for the effects of temporal changes in the *δ*^13^C value of atmospheric CO_2_ was applied by adding +2‰ [79], to facilitate comparison between modern and archaeological samples. This correction was also applied to modern reference material derived from other studies. Additionally, a lipid normalization calculation after Kiljunen et al. [80] was applied to all modern fish *δ*^13^C values.

Data evaluation was done using Microsoft Office Excel, PAST 4.03 and RStudio 5.01. Initial evaluation included the identification of outliers via Generalized Extreme Studentized Deviate as well as an assessment of normality via Shapiro-Wilk tests and of equal variance via *F*-tests. The two-tailed homoscedastic Student’s *t*-test was employed to assess any differences between groups, based on biological sex and burial cluster. The coefficient of variation (CV) was employed to compare variability between sites across the region. Differences between the median estimated dietary proportions in individuals with- and without available *δ*^13^C_apa_ measurements were assessed through a one-way PERMANOVA.

The amino acid fingerprinting method used available published *δ*^13^C_AA_ data from basal producers relevant to the site’s context, i.e., freshwater algae, freshwater cyanobacteria, terrestrial plants, bacteria, and fungi [36–38,81–96]. The analysis included *δ*^13^C_AA_ values of Leu, Ile, Phe, Thr and Val as EAAs most measured and associated with distinct values between basal groups. Subsequently both linear discriminant analysis (LDA) and principal component analysis (PCA) were conducted with values normalized through mean-subtraction and including the samples from YOO and Lake Onega. LDA is the more common approach in ecology studies [36,37,87] and provides a better distinction between basal groups, but PCA was applied as a more conservative approach due to it not taking prior (basal producer) group assignments into account.

Trophic positions were estimated using *δ*^15^N values of Glx and Phe and calculation of TPs followed Ishikawa et al. [20] as $TP=\frac{\delta^{\text{1}\text{5}}N_{Glu}-\delta^{\text{1}\text{5}}N_{Phe}+\beta }{TDF_{Glu}-TDF_{Phe}}+\text{1}$ with a TDF_Glu-Phe_ of 7.6‰ [97] and *β*_Glu-Phe_ of +8.4‰ reflecting the terrestrial and −3.4‰ the aquatic ecosystem [43,45,97]. As discussed above, both the *β* and TDF values are flexible, and while those used here are commonly referenced in archaeological studies [42,98,99], they represent averages, and other values have been suggested as well [46,100]. The *β* values also assume either fully aquatic or fully C3 terrestrial derived protein, which does not reflect the realities of human omnivorous behavior [42]. Accordingly, the calculated TP values provided in SI3, Table S2 in S1 Data, serve as rough estimates of the potential span of TPs occupied by humans, but should be considered guidelines rather than absolutes. Instead, the relationship between measured *δ*^15^N_Phe_ and *δ*^15^N_Glx_ values is considered the more meaningful proxy.

### Bayesian mixing models

Using the combined *δ*^13^C_col_, *δ*^15^N_col_ and *δ*^13^C_apa_ values FRUITS version 3.0 [101] was used to perform BMMs to estimate dietary source proportions at YOO. As with other BMMs, caution is warranted when interpreting FRUITS model outputs, particularly in aquatic dietary contexts, due to inherent limitations in baseline resolution and uncertainty regarding applied fractionation factors [19,101–103] as discussed in more detail below. To address these concerns, we implemented a series of sensitivity analyses using multiple model variants. The main model in FRUITS (model 1) was set up with three proxies (*δ*^13^C_col_, *δ*^15^N_col_ and *δ*^13^C_apa_), three sources (plant, game, fish) and three fractions (protein, energy – consisting of the lipids and carbohydrate portion – and bulk diet) following [21]. The contribution of protein to the *δ*^13^C_col_ signal was estimated at 74% ± 4% and energy at 26% ± 4% while the *δ*^15^N value is derived from dietary protein only (100% ± 0%) [21]. In the model the *δ*^13^C_apa_ signal is entirely derived from the bulk faction of the diet (100% ± 0%) [104].

A secondary model (model 2) considered only the contributions of dietary protein, using two proxies (*δ*^13^C_col_ and *δ*^15^N_col_) due to the restricted availability of *δ*^13^C_apa_ measurements. In the absence of *δ*^13^C_apa_ reflecting the bulk diet portion, only two fractions (protein, energy) were used, and the model was not concentration dependent.

Due to the lack of representative baseline *δ*^13^C_col_, *δ*^15^N_col_ and *δ*^13^C_apa_ values for the Lake Onega area, we decided to account for potential variations by running two model variants using slightly different baseline values. One employs only the values derived from YOO and Lake Onega to account for terrestrial animal and aquatic resources (models 1.1 and 2.1) and one incorporates a broader baseline spectrum including published values from multiple Fenno-Baltic and western Russian sites (models 1.2 and 2.2, for details see SI3, Table S3-S6 in S1 Data). The plant values employed were the same for both models, as no local values are available for plants from YOO, and included a wide geographical range of hemi-boreal, boreal, and subarctic samples. The source values were based on the weighted average of background data and adjusted to food values following [21] (SI3, Table S4 in S1 Data).

Trophic enrichment factors (TEF) in all models were set as *∆*^13^C_diet-coll_ +4.8 ± 0.5‰ and where applicable *∆*^13^C_diet-apa_ + 10.1 ± 0.5‰ [21]. TEFs for *∆*^15^N_diet-coll_ in the literature vary between ca. 3.0–5.5 ± 0.5‰ with the lower values more commonly applied in ecological and the higher in archaeological studies [103]. The drivers behind differences at this level are not entirely understood but appear to be related to both consumed diet and consumer (species) metabolism [15,105]. To account for this variability, we tested three different versions of the models using three different TEFs for ∆^15^N_diet-coll_ (a = 5.5‰, high; b = 4.6‰, medium and c = 3.6‰, low; [21,103,106] for detailed results see SI3, Table S6 in S1 Data). SD was maintained at 0.5‰ in all cases.

Macronutrient concentrations and standard deviations for the different dietary sources (SI3, Table S5 in S1 Data) were calculated based on values for dietary items likely available to past HGFs as given in [107], as well as studies on the total percentage of fat in prey animal bodies to account for differences between modern meat cuts and traditional use including, e.g., marrow and organs [108–113]. Prior information included in the model set the lower and upper limits for dietary protein contribution to 5–45% [101], based on physiological needs required to stay healthy and prevent protein poisoning [114].

## Results

### *δ*^13^C_col_ and *δ*^15^N_col_

The quality of newly measured archaeological *δ*^13^C_col_ and *δ*^15^N_col_ values was assessed via atomic weight C:N, with ratios between 3.0–3.4 accepted as representing sufficiently well-preserved collagen [115,116] (SI3, Table S1 in S1 Data).

One low outlier was identified in both *δ*^13^C_col_ (burial 59 at −21.5‰, R_1 _= 3.44, λ_1 _= 3.20, α = 0.05) and *δ*^15^N_col_ (burial 57 at 12.5‰, R_1 _= 3.54, λ_1 _= 3.21, α = 0.05) (SI2, Fig S3 in S1 File), which were subsequently omitted from statistical evaluation, including mean values. Afterwards, both *δ*^13^C_col_ and *δ*^15^N_col_ values did not depart significantly from a normal distribution (Shapiro-Wilk *W*-test: *δ*^13^C_col_ *p *= 0.0874; *δ*^15^N_col_ *p *= 0.9926). Adding the new human values to those of Wood et al. [2] and Schulting et al. [1] provided an overall average of −20.0 ± 0.4‰ (n = 59) for *δ*^13^C_col._ The new dentine-based measurements were slightly lower (−20.2 ± 0.4‰, n = 19) than the bone collagen values (−19.9 ± 0.3‰, n = 40) by a small but statistically significant margin (Student’s *t*-test, *p *= 0.0172). The new dentine *δ*^15^N_col_ values (average = 16.5 ± 0.7‰, n = 19) were similarly slightly different from the bone collagen values (16.0 ± 0.8‰, n = 41; Student’s *t*-test, *p *= 0.0207), resulting in a new overall average of 16.1 ± 1.0‰ (n = 59) (SI2, Fig S3 in S1 File). Considering the consistency in the measurements of standards between both laboratories (SI1, Table S1 in S1 File), these minimal differences are most likely a result of the different age stages captured by the sampled tissues and will be discussed further in an upcoming publication (Eckelmann et al., in prep).

**Fig 2:**
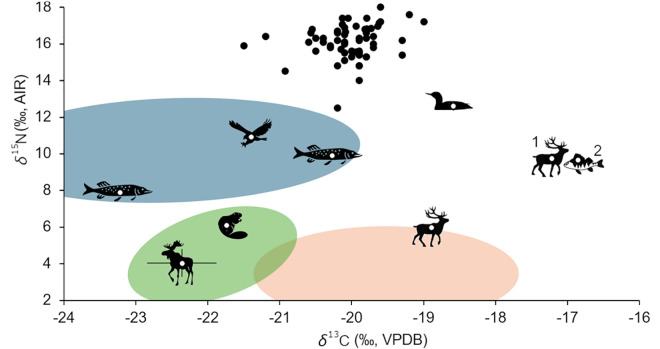
Summary of available δ^13^C_col_ and δ^15^N_col_ data from YOO and Lake Onega. Human (black circles) and faunal (symbols) values from YOO are plotted in front of 65% ellipses for freshwater fish (blue), reindeer (red) and other terrestrial animals (green) from the wider geographic supra-region included in dietary model 2 (see SI3, Table S3 in S1 Data for all baseline isotope values included). YOO faunal values, except for reindeer, modified from Schulting et al. [1] including a different (+2‰) adjustment for changes in *δ*^13^C value of atmospheric CO_2_ of modern data. One reindeer is likely affected by a suckling effect (1). Perch samples were omitted from dietary modelling due to anomalous values, except for one individual that was also sampled for CSIA (2) to facilitate comparison between CSIA and bulk collagen isotope values.

Overall, the human data were narrowly distributed (Fig 2) and even if outliers were included, variation was low (*δ*^13^C_col_ CV = 2.2%; *δ*^15^N_col_ CV = 5.7%). This translated into a continued absence of any significant difference between the sexes, as previously reported by Schulting et al. [1], taking into consideration the new isotopic data and updated sex identifications (Student’s *t*-test *δ*^13^C_col_ *p *= 0.9700, *δ*^15^N_col_ *p *= 0.4932) (SI2, Fig S4 in S1 File). Similarly, no significant difference between the North and South sections of the cemetery was found (Student’s *t*-test, *δ*^13^C_col_ *p *= 0.0825, *δ*^15^N_col_ *p *= 0.0534) (SI2, Fig S5 in S1 File).

The new faunal analyses revealed unusually high *δ*^13^C_col_ values of Onega perch, from −17.5 to −11.8‰ after lipid-correction (SI3, Table S3 in S1 Data). While there are some instances of similarly high values known from freshwater fish in other areas, [117], none have been found in Finnish lakes [65,118–120]. Fish from Finnish lakes usually show values of ca. −25 to −19‰ (ibid.) and, as they are in similar environments, they should be expected to be at least broadly comparable isotopically to Lake Onega. Further investigation revealed a recent substantial shift in perch feeding ecology in Lake Onega, due to the lake’s colonization by the Baikal amphipod *Gmelinoides fasciatus* [121]. This invasive species now provides up to 80% of the diet of young perch [122], likely indicating a shift in foraging behavior towards its prey’s littoral habitat. As this shift is recent and may have affected *δ*^13^C_col_ values we decided to exclude the perch values from the FRUITS models.

The two measured archaeological reindeer samples exhibited relatively high *δ*^13^C_col_ and *δ*^15^N_col_ values (average *δ*^13^C −18.1 ± 0.8‰, *δ*^15^N 7.8 ± 1.9‰), with one individual’s *δ*^15^N_col_ being unusually high at 9.7‰. Considering that the value was measured on the root of a first molar and the next highest Holocene reindeer *δ*^15^N_col_ values are ca. 8.5‰ [123,124], this potentially derives from a suckling effect. The second value at 5.9‰ was measured on a third molar and is therefore not affected by a suckling effect, being in line with other Fennoscandian reindeer values [125,126].

Overall, the initial assessment of the baseline information indicates a substantial difference between human *δ*^15^N_col_ values (16.6 ± 0.7‰) and the assumed trophic baseline, with the highest value from Lake Onega being the zander measurement at 9.9‰. This offset exceeds both the assumed trophic offsets of ca. 3.0‰ commonly applied in ecology studies, as well as the 5.5‰ often used in archaeological studies [103] when considered against the strictly local baseline values. It is also within the trophic offsets’ upper limit when compared to the baseline data for the wider supra-region used in dietary model 2 (average ca. 10.3 ± 2.3‰; SI3, Table S3 in S1 Data). Although waterbirds do have a tendency towards higher values, as demonstrated also by the *δ*^15^N_col_ of the black-throated diver and the osprey previously analyzed from YOO [1], and were surely hunted, they would have only been available seasonally, though this could have been extended if kept in a storable form.

### *δ*^13^C_apa_

There are no firmly established quality criteria for the preservation of carbon isotope values in enamel apatite, but enamel is by far the best-preserved skeletal tissue, being more resistant to diagenesis than bone and results derived from it widely accepted as reliable [127,128]. Similarly to the bulk collagen isotope values, the *δ*^13^C_apa_ measurements showed a limited variability between samples with a mean for the sampled individuals of −15.5 ± 0.4‰ (see SI2, Fig S3 in S1 File). The *Δ*^13^C_apa-coll_ spacing averaged 4.8 ± 0.4‰, consistent with that expected for a tissue offset, given isotopically homogenous protein and energy fractions in the diet [129–131].

### *δ*^13^C_AA_ and *δ*^15^N_AA_

All six archaeological human and three modern fish samples analyzed for *δ*^13^C_AA_ and *δ*^15^N_AA_ passed general quality control criteria. However, no measurements were reported for methionine as it fell below the detection limit, or for δ^15^N_Tyrosine_ which was also below detection for the archaeological samples and showed poor peak separation for the modern samples. Details on quality controls and results for the individual amino acid measurements are provided in supplementary information (SI1; SI2, Fig S1-2 in S1 File; SI3, Table S2 in S1 Data).

As with the bulk collagen values, *δ*^13^C_AA_ and *δ*^15^N_AA_ results showed a high degree of similarity among the human individuals (average SD 0.4‰ for *δ*^13^C_AA_; 1.3‰ for *δ*^15^N_AA_), whereas the three measured fish exhibited a greater variability (average SD: 1.1‰ for *δ*^13^C_AA_; 2.0‰ for *δ*^15^N_AA_). Even after excluding the perch, which similar to its *δ*^13^C_col_ had substantially higher *δ*^13^C_EAA_ values than the other fish and humans, the two remaining fish retained a wider range of observed values than the humans. Human *δ*^13^C_EAA_ closely resembled those of the zander, and to a lesser extent the vendace, with divergence in the *δ*^13^C_NEAA_ values remaining below 3‰. In *δ*^15^N_AA_, zander and vendace varied in *δ*^15^N_AAtr_ values, which is expected as zander are fully carnivorous and vendace are mixed feeders. Human *δ*^15^N_AAtr_ were mostly aligned with those of the zander. *δ*^15^N_AAsrc_ values were broadly similar across species for *δ*^15^N_Lys_ values but showed notable interspecies differences for *δ*^15^N_Phe_ (on average by 3.7‰).

The human *δ*^13^C_Glx_ values were relatively low (−28.9 ± 0.1‰), if not the lowest measured to date on an archaeological human population; conversely, the corresponding *δ*^15^N_Glx_ values (*δ*^15^N_Glx _= 26.0 ± 1.1) were at the high end of reported values from archaeological human samples with the lowest value from YOO being 24.8 ‰. Similarly high or higher values so far originate overwhelmingly with coastal populations [98]. In general, *δ*^15^N_AAtr_ values were high compared to other known data, which also applied to *δ*^15^N_Lys_, *δ*^15^N_Ser,_ and *δ*^15^N_Gly_. *δ*^15^N_Phe_ was an exception with a more common range of 3.7–5.6‰. This resulted in a high *δ*^15^N_Glx-Phe_ offset at 21.0 ± 1.4‰ (n = 6) for YOO, compared to, e.g., 22.9 ± 2.4‰ (n = 11) for marine mammal hunters in northern Japan [98], and 18.3 ± 0.9‰ (n = 2) and 17.4 ± 0.9‰ (n = 2) for Mesolithic coastal HGFs in Croatia [132] and Scotland [32]. The geographically closest measurements from a medieval inland site in Finland by comparison only had values of 7.9 ± 2.2‰ (n = 5) [133].

The *δ*^13^C_AA_ fingerprinting approach was attempted, but while both PCA1 (86.6% of the variation) and PCA2 (7.9% of the variation) were partially successful at separating the basal producer groups, the overlap between freshwater components, fungi and bacteria remained high (SI2, Fig S6 in S1 File). The situation was similar with the LDA, indicating high discrimination in the first and second axis (80.4% and 16.1% respectively) and a better spatial differentiation, but only 74.5% of values were successfully re-categorized in the confusion matrix due to the high overlap between freshwater producers and the other categories (SI2, Fig S7 in S1 File) [36]. While we did therefore not consider the separation between groups sufficient to attempt percentage assessments of dietary contributions from each of these variables to the YOO diet, the placement of both YOO human and the Lake Onega fish samples on the graphs is of some interest. First, in both analyses the YOO humans and Lake Onega fish plot closely together indicating a very high degree of similarity in their essential amino acid profiles, and thus, protein sources. Their tight clustering is consistent with humans sourcing the majority of their proteins from the same trophic chain as the Lake Onega fish. Second, both fish and humans plotted separately from the terrestrial plants and showed a higher degree of similarity with bacteria and, to a lesser degree, fungi and freshwater components. If we were only looking at the human values, this could indeed be interpreted as a potential indicator of fungal consumption. However, as the fish showed very similar positioning, it is more likely that these values are indicative of input of allochthonous litter and nutrient cycling through the Lake Onega hydrological system, rather than of direct dietary intake.

### FRUITS dietary estimate results

The results obtained both for individuals and the overall average for YOO did not vary substantially between the model variants using different trophic offsets and applying restricted or broader baseline values and overall relation of dietary sources was maintained (Table 2, complete output in SI3 Table S6 in S1 Data). This general consistency between models indicates that they do not significantly affect results in our case and that the model estimates are robust, within the parameters applied. Standard deviations were small, and model convergence as estimated through FRUITS-derived Markov chains was acceptable [21,34].

**Table 2:** Comparison of results for the different applied FRUITS dietary mixing models. Estimated contributions of source fractions to the complete diet and protein portion, respectively, according to FRUITS models based on the average *δ*^13^C_col_, *δ*^15^N_col_ and *δ*^13^C_apa_ values at YOO and varying according to the inclusion of either game/fish values only from Lake Onega (1.1, 2.1) or from a broader geographical range including Finland and north-western Russia in the source values (1.2, 2.2), and to varying TEF values for *δ*^15^N_col_ (a = 5.5‰, b = 4.6‰, c = 3.6‰).

Bulk Model 1	1.1a	SD	1.1b	SD	1.1c	SD	1.2a	SD	1.2b	SD	1.2c	SD
Plants	0.54	0.04	0.54	0.04	0.53	0.04	0.56	0.06	0.56	0.05	0.54	0.04
Game	0.04	0.03	0.03	0.02	0.02	0.02	0.07	0.06	0.01	0.01	0.04	0.04
Fish	0.42	0.05	0.44	0.04	0.45	0.04	0.37	0.07	0.42	0.05	0.41	0.05
Protein Model 1	1.1a	SD	1.1b	SD	1.1c	SD	1.2a	SD	1.2b	SD	1.2c	SD
Plants	0.22	0.03	0.22	0.03	0.21	0.03	0.23	0.05	0.23	0.04	0.22	0.03
Game	0.06	0.06	0.05	0.04	0.03	0.03	0.12	0.09	0.03	0.02	0.07	0.06
Fish	0.72	0.06	0.74	0.05	0.75	0.04	0.65	0.10	0.74	0.05	0.70	0.07
Protein Model 2	2.1a	SD	2.1b	SD	2.1c	SD	2.2a	SD	2.2b	SD	2.2c	SD
Plants	0.07	0.06	0.05	0.04	0.03	0.03	0.10	0.07	0.08	0.07	0.07	0.06
Game	0.14	0.11	0.09	0.07	0.06	0.05	0.23	0.16	0.16	0.13	0.11	0.10
Fish	0.79	0.11	0.86	0.08	0.91	0.05	0.67	0.15	0.76	0.12	0.81	0.10

**Fig 3:**
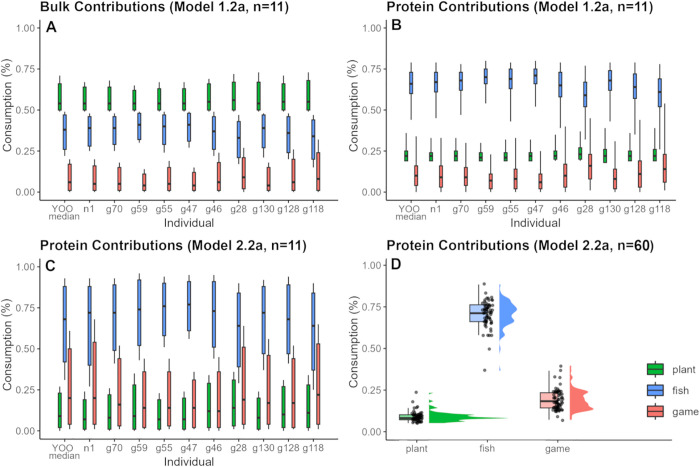
Summary of results of applied FRUITS dietary mixing models for multiple individuals from YOO. Estimated dietary proportion of fish, game and plants to the YOO diet for mean values (first value in graph A-C) and the ten individuals for which apatite *δ*^13^C values were available. A) Model 1.2a indicating estimated contribution of different food sources to bulk dietary intake. B) Contribution of protein based on model 1.2a using combined collagen and apatite measurements. C) Estimated contribution of protein based on model 2.2a only using collagen data shown for the same eleven individuals represented in A and B. D) Contribution of protein based on *δ*^13^C_col_ and *δ*^15^N_col_ summarized for the complete data set.

Bulk diet model 1, based on combined *δ*^13^C_col_, *δ*^13^C_apa_ and *δ*^15^N_col_ data, estimated that for the average individual at YOO ca. 55% of dietary intake was derived from terrestrial plants, ca. 40% from aquatic resources and only ca. 5% from terrestrial game (Fig 3A). It also estimated the contribution to the *δ*^15^N signal, signifying the protein portion, as deriving ca. 70% from aquatic resources, ca. 20% from terrestrial plants and only ca. 10% from terrestrial game. (Fig 3B) This deviates from model 2, using only *δ*^13^C_col_ and *δ*^15^N_col_ to assess protein contributions, which indicated a higher contribution of meat than plants to the protein signal (Fig 3C-D). However, as model 2 had fewer proxies, higher standard deviations and lower model convergence, the model 1 results are likely more reliable from a modelling perspective, although this demonstrates that overlap between the estimates for plant and game contributions exists. The overall isotopic variation between individuals is very restricted, the ten individuals for which *δ*^13^C_apa_ values were available span the range of that restricted distribution, and the estimated dietary proportions in model 2 do not diverge between individuals with and without apatite values (One-way PERMANOVA, *p* = 0.5074). Therefore, we consider the results from model 1 as representing the overall community at YOO.

## Discussion

### YOO and the wider Early Holocene (Northern European) HGFs

**Fig 4:**
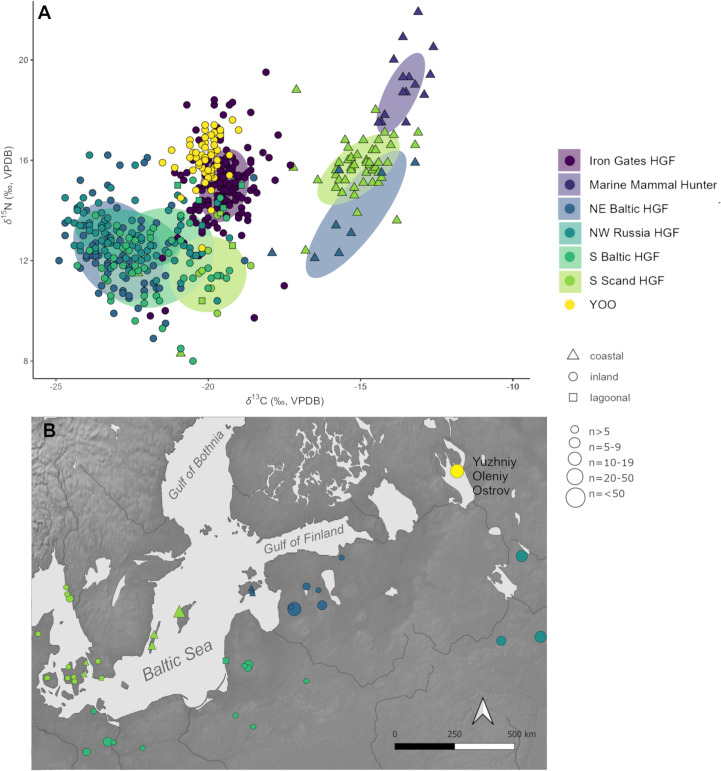
Overview of δ^13^C_col_ and δ^15^N_col_ values from YOO and other HGF sites in the northeast Baltic. A) *δ*^13^C_col_ and *δ*^15^N_col_ values from YOO and other archaeological HGF sites (65% confidence for ellipses, separated for inland and coastal groups) focused on northeastern Europe clearly showing the unusually high *δ*^15^N_col_ values of the YOO population compared to other boreal inland HGFs. Values from marine mammal hunters (Greenland and Hokkaido) and the Mesolithic of the Danubian Iron Gates region (Romania and Serbia) are included for comparative purposes [2,40,63,98,136–170]. B) Spatial distribution and sample size of the groups and sites included in A (for details see SI 2, Fig S8 in S1 File) [51]. The site map was created using Natural Earth public domain data [52].

Within the broader dataset of archaeological HGF communities of the circum-Baltic region for which *δ*^13^C_col_ and *δ*^15^N_col_ values are available, YOO forms a distinct isotopic cluster. This is characterized by notably high *δ*^15^N_col_ values, comparable to communities with a substantial marine diet, and *δ*^13^C_col_ values that, while still situated in the range of known freshwater contexts in this region, are higher than the majority of other known inland HGF sites of the eastern Baltic and north-western Russia (Fig 4). The higher *δ*^13^C_col_ values, while not as unusual as the *δ*^15^N_col_ values, are still unexpected, as the other comparable sites in the north-east European forest zone are located south of YOO and in general carbon isotope values would be expected to decrease in higher latitudes [134,135].

Generally, the *δ*^13^C_col_ and *δ*^15^N_col_ values with the most consistent similarity to YOO are not those from the north-western Russian and eastern Baltic HGF sites geographically closest, but from the Mesolithic of the Danubian Iron Gates region (Romania and Serbia). This archaeological culture is known for its high reliance on freshwater and anadromous species and the significance of fish in their material culture and iconography, which is unique in Europe [144,171–173]. While it is expected that the YOO population also relied heavily on aquatic resources, as an effect of their importance in the local landscape and by comparison with modern HGF of the boreal forest zone, the majority of the other aforementioned Baltic and Russian sites are similarly found in aquatic landscapes. As they are also located in the northeast European forest zone, the expectation would have been for their isotope ecologies to be more in line with those of YOO, compared to the Iron Gates region.

When viewed in the geographical context of north-western Russia and the circum-Baltic, the values most similar to YOO originate either from children potentially still affected by nursing signals (e.g., Zamostje in Russia, Kivisaare in Estonia) [63,167] or lagoonal sites (e.g., Šventoji in Lithuania and Skateholm in Sweden), which had marine contributions to the diet, especially in the form of seals [151,159]. Since the values from YOO reflect post-weaning values, of which two thirds were derived from adult bone collagen, the nursing effect is not what is driving the position of the YOO cluster. Instead, it is likely that the local isotope ecology of the Lake Onega area differs significantly from other sites of the northeast European forest zone represented here, leading to substantially higher human *δ*^15^N_col_ and slightly raised *δ*^13^C_col_ values. However, this is not visible in the admittedly very small local faunal sample, whose isotope values are largely comparable with values from other sites in this ecozone.

Considering the similarity of the YOO values to the lagoonal sites, especially Šventoji, two subsistence-based scenarios could be considered as causing the observed elevations. Firstly, that dietary items with marine or mixed freshwater-marine signals contributed at least periodically to the YOO diet. This may have occurred as a result of periodic trips to the coast (either the Baltic or White Sea, see Fig 1), exchange of goods, or through hunting of migratory birds. Anadromous fish, which contributed to the isotopic fingerprint of the Iron Gates Mesolithic [144], are not considered relevant to this study, as the migratory fish species in Lake Onega are estimated to have been landlocked since at least 7500 BCE [174].

The second possibility for explaining the distinct position of YOO is the potential that higher *δ*^15^N_col_ and *δ*^13^C_col_ values could have been acquired through hunting freshwater seals, as are found today in Lakes Saimaa (*Pusa saimensis*) and Ladoga (*Pusa hispida ladogensis*) and might have once also been present and hunted in Lake Onega. These seals are the local freshwater apex predators in their ecosystems and have consequently elevated *δ*^15^N and to a lesser degree *δ*^13^C values compared to their prey [120]. This latter scenario appeared particularly plausible as samples from Lake Saimaa freshwater ringed seals neatly slotted into a perceived gap in the *δ*^15^N value distribution between available fish and human values for Onega and its surroundings [1].

*δ*^13^C_col_ and *δ*^15^N_col_ values alone, particularly lacking access to a broad local (aquatic) baseline, are not able to resolve these questions, but with the addition of the new *δ*^13^C_apa_ and CSIA results presented here, it is possible to narrow down the most plausible contributing factors and their subsistence implications. Accordingly, both scenarios will be discussed in detail in the following sections, as well as potential alternative explanations.

### No isotopic evidence for marine dietary input

In principle, even though the observed YOO *δ*^15^N_col_ values are surprisingly high, they could be acquired with no marine input if a trophic offset between human consumers and diet at the higher end of the observed range is assumed and dietary protein was primarily derived from high in the aquatic food chain. However, ethnographic sources clearly indicate that HGFs can and in some cases do regularly transverse large distances in the course of seasonal moves [175–177]. Considering the presence of major rivers leading both north- and westwards, this could be considered a possibility for the YOO population as well and therefore the presence of marine dietary input cannot be ruled out without further assessment.

Additionally, it could also be possible to acquire very high *δ*^15^N_col_ values more in line with expectations for marine ecosystems through intensive seasonal fowling of migrating waterbirds, e.g., as Cree communities in northern North America are known to practice [178]. Zooarchaeological finds attest to whooper swan (*Cygnus cygnus*) hunting at Mesolithic sites in Denmark [179,180]. Some of the bird species found at YOO and other Onega sites spend a substantial time of the year in marine habitats and accordingly can have more marine values even if caught in freshwater settings (e.g., the black-throated diver) from YOO with *δ*^13^C_col_ −18.6‰ and *δ*^15^N_col_ 12.6‰, [1]. The effect of such a partial marine signal might also contribute towards the observed slightly elevated carbon isotope values.

**Fig 5:**
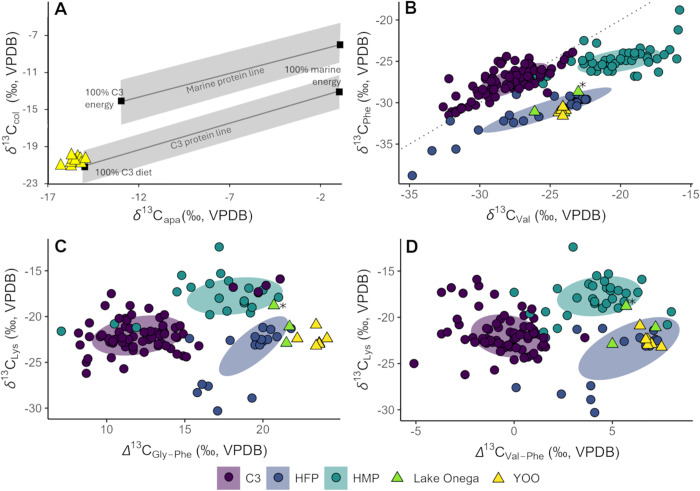
Overview of δ^13^C_col_, δ^13^C_apa_ and δ^13^C_AA_ used to assess origin of dietary sources at YOO. A) The relationship between human *δ*^13^C_apa_ and *δ*^13^C_col_ values from YOO plotted against diet-specific regression lines after Froehle et al. [28] (following [186]). B-D) Bivariate plots of B) *δ*^13^C_Phe_ vs. *δ*^13^C_Val,_ C) *δ*^13^C_Lys_ vs. *Δ* ^13^C_Gly-Phe_ and D) *δ*^13^C_Lys_ vs. *Δ*^13^C_Val-Phe_, showing the values from YOO humans and Lake Onega fish compared with published data from humans and animals with known dietary background data (65% confidence ellipses) [35,40,42,132,139,169,187]. The perch with anomalous bulk *δ*^13^C_col_ values (see Results) potentially associated with an altered feeding ecology is marked with an asterisk.

To try and clarify this matter, we employed *δ*^13^C_apa_ and *δ*^13^C_AA_ ratios, both of which can also serve as more sensitive marine tracers than *δ*^13^C_col_ and *δ*^15^N_col_ values and confirm that an isotopically visible impact of marine sources to the YOO diet is highly unlikely. Experimental studies indicate that the relationship between *δ*^13^C_col_ (reflecting primarily the protein portion) and *δ*^13^C_apa_ (reflecting the complete dietary mix) forms two regression lines that reflect the origin of the consumed proteins and allows an assessment of the contribution of C3 and marine sources to the whole diet, rather than just the protein portion [28,29,181]. In the case of YOO (Fig 5a), this model is compatible with a fully C3/freshwater-based diet and indicates no relevant marine contribution. However, a caveat is that there is increasing evidence for a systematic offset between *δ*^13^C values of bone apatite and enamel apatite [182–184], which complicates the application of these models to our data as the models were based on bone apatite measurements [144]. The available evidence suggests that enamel apatite carbon isotope values are enriched in ^13^C compared to bone by ca. 0.6–1.8‰ [182–185] but the effect is not yet well understood.

The *δ*^13^C_AA_ values similarly support a largely freshwater based isotope signal. There are multiple approaches to using *δ*^13^C_AA_ to differentiate marine, freshwater and terrestrial signals, e.g., different metrics and statistical methods [23,30,38,87,169]. We chose to use a comparative approach common in archaeological studies [32,103,169], in which the results from YOO are compared to data from other populations with known dietary backgrounds. The proxies chosen for this comparison were *δ*^13^C_Val_, *δ*^13^C_Lys_ and *δ*^13^C_Gly_, plotted against *δ*^13^C_Phe,_ *Δ* ^13^C_Gly-Phe_ and *Δ*^13^C_Val-Phe_ respectively. The former are all tracers distinguishing between aquatic and terrestrial resources, and in the case of *δ*^13^C_Val_ also between freshwater and marine resources, while the latter remain largely unaffected by trophic effects [39,40,169]. The results of these comparisons show that the YOO humans were largely consistent with other consumers of primarily freshwater-derived protein rather than with marine consumers (Fig 5). It is interesting to note that the modern perch from Lake Onega with anomalous bulk *δ*^13^C_Col_ has *δ*^13^C_Lys_ values trending towards the marine range, providing another indicator of the isotopic plasticity of freshwater systems, especially those as complex as that of Lake Onega.

Overall, these results show that there is currently no convincing stable isotope evidence for detectable marine contributions to YOO diet, whether from migrating waterbirds or through human mobility. This is supported by recently published ^87^Sr/^86^Sr values which also indicate that the population probably permanently resided in the wider northern Onega area [66], and travel to either the White or the Baltic Sea from Lake Onega during the Late Mesolithic was unlikely to have been a regular occurrence. More likely, the slightly elevated *δ*^13^C values are endogenous to at least parts of the Lake Onega hydrological system, potentially influenced by the size of the lake. Large lakes can be relatively ^13^C-enriched as a result of more efficient exchange with atmospheric CO_2._ Another potential source of enrichment is carbonate bearing bedrock underneath the lake [188,189]. Moreover, freshwater systems generally exhibit a high degree of isotopic plasticity [190].

### No seals required to explain observed *δ*¹⁵N

The second suggested explanation for the relatively high *δ*^15^N_col_ values is the potential hunting of freshwater seal, based on the existence of remnant seal populations trapped in freshwater lakes due to post-glacial land uplift in the early Holocene. Today there are still populations remaining in Lake Saimaa (*P. saimensis*) in Finland and Lake Ladoga (*P. hispida ladogensis*) in Russia, and at least the latter was intensively hunted during the Stone Age [191,192]. While there is no modern population in Lake Onega, the question of whether one existed and became extirpated has been posed by both biological and archaeological studies [62,193], especially when the first *δ*^13^C_col_ and *δ*^15^N_col_ values from YOO emerged [2,63]. Even though comparative *δ*^15^N_col_ values from Lake Saimaa would place seals at the perfect position in the food web (12.4 ± 1.0‰, n = 23 [1,120]) to close the perceived trophic gap between measured human (16.1 ± 0.1‰) and the small number of available present-day fish *δ*^15^N_col_ values (highest at 9.0‰) from Lake Onega, it is highly unlikely that a seal population (ever) existed in Lake Onega. While finds of seal bone have been reported, these currently amount to only two instances, comprising three bone fragments from Kladovets II [194] and two bones from Fofanovo XIII [195]. If compared to the prevalence of seal bone finds in the Ladoga area, where seal makes up about half of all identified mammalian bones [191,192], this is not convincing evidence for the presence of a local seal hunting tradition – although the number of fully investigated faunal assemblages from Onega is substantially smaller. In their palaeogeographical investigation, Ulichev and Ludikova [193] further concluded that a penetration of seal into Lake Onega through Lake Ladoga was improbable, citing the lack of an appropriate corridor facilitating this movement, given that Lake Onega is part of a different hydrological paleobasin than both the Baltic and Lake Ladoga [193]. The *δ*^15^N_AA_ results confirm that seal are not necessary to explain the values observed in the archaeological human population, but that the unusually high *δ*^15^N values are likely related to shifts at the aquatic baseline levels of Lake Onega.

**Fig 6:**
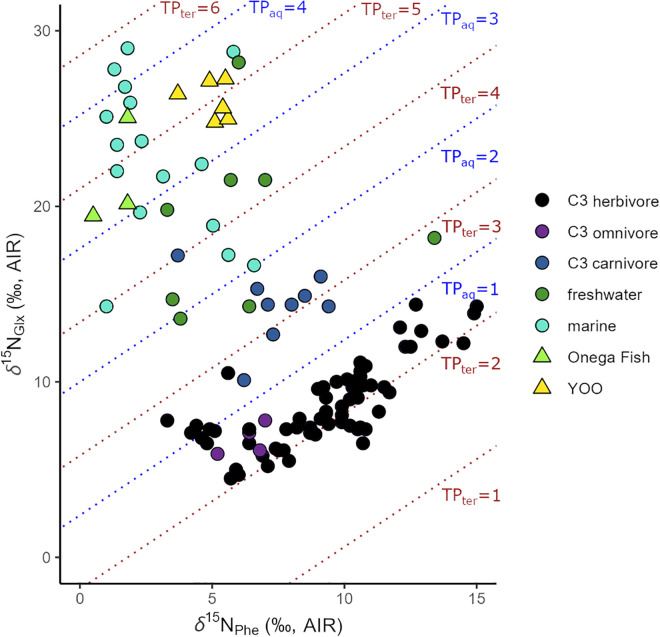
δ^15^N_Glx_ against δ^15^N_Phe_ from collagen of YOO humans and Lake Onega fish. The blue and brown dotted lines mark trophic position estimates in the aquatic (blue) and terrestrial (brown) food chain [78,98]. They, as well as the animal references from a global background [32,35,49,98,132,196,197] should be considered as guidelines only [78].

As significantly more precise indicators of trophic relationships, the *δ*^15^N_AA_ values we analyzed show that while bulk *δ*^15^N_col_ for the Lake Onega fish are substantially lower than those of the human population (Fig 2), their *δ*^15^N_Glx_ and *δ*^15^N_Phe_ values place the YOO humans above the mixed feeders vendace and perch (the latter with possibly non-analogous feeding ecology), and at a comparable level to the predatory zander (Fig 6).

Thus, if the Mesolithic Lake Onega supported similar stocks of fish with a broadly comparable trophic structure, the trophic position of the YOO humans could have been attained by fish consumption.

While it is not possible to completely exclude the existence and consumption of seals at Lake Onega, the consequence of the previous assessments on the likelihood of seal ever having been present locally – which is low – with these values, is that there is currently no reason to believe seal was ever part of a regular subsistence strategy at the site or should be considered in the FRUITS modelling. Individuals might have occasionally had access to seal through human mobility and/or the exchange of seal meat, as evidenced by the rare finds of seal bones, but freshwater fish and aquatic bird consumption sufficiently explains the observed bulk *δ*^15^N_col_ signal. Metabolic factors [198] or food processing, could have potentially further raised the values, e.g., preservation through fermentation, which is a common practice in many northern cultures and known to be practiced in the Mesolithic [199], has been observed as increasing *δ*^15^N values by ca. 1.3‰ [200].

It should be noted that both the offset in bulk *δ*^15^N_col_ values between present-day fish and YOO humans and the *δ*^15^N_AA_ values suggest that modern Lake Onega may not be representative of the past in terms of *δ*^15^N baselines. The difference in *δ*^15^N_Phe_ values between modern fish (1.4 ± 0.6‰) and ancient YOO humans (5.0 ± 0.6‰) shows access to different nitrogen pools, either due to temporal shifts in the Onega ecosystem or different feeding regimes. While not affecting the trophic relationships as seen through the *δ*^15^N_Glx_ to *δ*^15^N_Phe_ proxy, this indicates that the bulk isotope measurements of the modern fish likely do not represent the ecosystem of Lake Onega during the Late Mesolithic, which would make them less suitable for use in the FRUITS model. The ecology and structure of the lake’s fish populations are likely to have been severely impacted by recent overfishing. Nevertheless, as the results of the models using the values from Lake Onega only (model 1.1 and 2.1) and the wider baseline approach (model 1.2 and 2.2) do not differ substantially from each other, the effect on the dietary reconstructions appears to be minimal.

### Subsistence and dietary reconstructions

Regarding the protein portion of the diet, the results of the FRUITS models employed in this study suggest that freshwater fish (or other resources with a similar isotopic signal, e.g., waterbirds) were the dominant source at YOO with only a small contribution from terrestrial game (Table 2, Fig 3B–D). While not reflected in the material culture of the cemetery [52], a high contribution of aquatic resources to the protein intake is in line with expectations for boreal HGF cultures, and findings at other Mesolithic sites [63,152,158,167,168,201]. However, even though fish as the main source of protein is a common assessment both ethnographically and archaeologically, the low estimated contribution of hunted game (12 ± 9%) and relatively high suggested plant intake (23 ± 5%), even in the protein component of diet, do not conform with general notions of boreal HGF subsistence [201]. This problem is even more apparent in the estimated contributions of the three food sources to total dietary intake. By proposing that on average ca. 56% of the diet was derived from plants, the modeling results put a spotlight on a longstanding debate in archaeological research concerning both the overall importance of plant foods in pre-agrarian societies and the pitfalls of dietary reconstructions via mixing models. On one hand these results (plant food contribution at 56 ± 6%, fish at 37 ± 7% and game at 7 ± 6%) show a large degree of coherence between the different models we applied as well as with estimates at other north-eastern European inland HGF sites. While comparisons between different BMMs should always be treated with caution, similar proportions indicating dietary contributions of ca. 50–60% plants were estimated from early Holocene HGF remains at Narva Joaorg I, Veibri, and Tamula in Estonia [167], the younger Mesolithic phase at Zamostje in north-western Russia [63] and the earliest phases of Zvejnieki in Latvia [152]. On the other hand, these results deviate substantially from ethnographic records describing the dietary importance of different food sources in HGF societies, in which plant contribution in the boreal zone and higher latitudes is generally described as not exceeding ca. 25% [201,202]. The accuracy of these figures for the gathering component in many (especially historical) ethnographic reports has been extensively questioned, due to the prevalence of issues such as observer or reporting bias (e.g., due to gendered working patterns and male ethnographers interacting primarily with men, or a focus on more prestigious activities) [203–205]. Yet, the fact remains that a large divergence of 50–60% estimated and 25% described plant consumption should have impacted daily life sufficiently to be notable to observers. This raises the question of, whether, in contrast to usual concerns [206], BMMs may not have overestimated the dietary contribution of plants (see also [102]).

Relatively low plant consumption observed in modern and historical boreal HGFs are argued to be partially caused by the overall low plant productivity and strong seasonal restriction in the boreal forest zone, compared to more temperate environments, leading to an increased reliance on animal, and especially aquatic, resources [201]. However, it is also true that plant resources are chronically underestimated and understudied in hunter-gatherer research [207–209] and while lower in productivity than other ecosystems, boreal forest environments do produce seasonally bountiful crops, especially of berries and mushrooms. While mushrooms are surprisingly often absent as a food source in ethnographic records, fungi are an important resource in historical foraging across northern Europe produce bumper crops, are easily preserved and contain relatively high amounts of protein (10–35% dry weight according to [210]). The latter has seen them previously suggested as potentially impacting stable isotope values of consumers, specifically as raising *δ*^15^N values [211], though so far there has not been much success in verifying their consumption via stable isotope analysis in archaeological samples. CSIA fingerprinting [37] could potentially help to fill this gap, but the attempt made in this study remains inconclusive due to an overlap of source values. Berries, however, can be easily harvested in large quantities and constitute a substantial part of wild foods consumed in most records for the boreal forest zone. Reportedly, 70–100 kg of lingonberries (*Vaccinium vitis-idaea*) could be harvested within a day in good berry patches of Karelia in the middle of the 20^th^ century [210,212] and modern Gwich’in women in northern North America reported harvesting ca. 12 L of different berries within a 3–4 hour harvesting event [213]. Wild tubers, tree cambium and some types of edible lichen may be less prominent but are often accessible in a wide seasonal window and have a long history of use, in addition to many other plants [209,210,214–219]. Tuber starch specifically has been previously identified in dental calculus at YOO [220]. Buckley and colleagues [221] further identified the widespread use of freshwater plants in the European Stone Age, many of which are also found in Karelia, e.g., waterlily (*Nymphea* sp.) and bulrush (*Typha* sp.*)* [222], and could have contributed to diet. The YOO population apparently also exhibits a high incidence of caries (> 20%) (Zubova et al., in prep.) which has often been connected to carbohydrates in the diet, although that connection remains contested (for review see [223]).

Given these observations, it is not hard to imagine that plants did indeed play a larger role in prehistoric YOO subsistence than would be expected from most ethnographic accounts and a research tradition more focused on hunting and fishing. Nevertheless, we still consider it unlikely, that the plant component would be consistently underreported by more than half of total consumption, which would be necessary to account for values as high as the 57 ± 6% estimated for YOO. Even the highly effective Gwich’in berry harvesters are estimated at only providing ca. 5% of a household’s overall traditional diet with game and fish being substantially more important [224].

One point supporting the possibility that our FRUITS models have overestimated plant consumption is the human *δ*^13^C_EAA_ values, both in their substantial separation from the plant values in PCR and LDA (SI1, Fig S6 – 7 in S1 File), and their high similarity between humans and fish, specifically the zander. Even considering the fact that the modern fish are less than ideal proxies for prehistoric ones, it seems unlikely that an estimated 30–35% of protein originating from terrestrial sources, i.e., game and plants in the human diet (see Table 2) would not lead to a stronger divergence between human and fish *δ*^13^C_EAA_ values.

Ultimately, while our models are internally coherent and their results align with findings from other sites under similar conditions, they remain constrained by the parameters provided. A key limitation, for instance, is the lack of local plant baseline values appropriate to the Mid-Holocene Lake Onega region, as well as the limited taxonomic range of sampled fauna. Nevertheless, the similarity of the few existing YOO faunal values (perch aside) to those from the wider region suggests they are likely representative. However, a major driver of the high plant estimates in the model is the prior restricting protein intake to 5–45% [101]. If this prior is disabled, the estimated plant contribution is almost halved (to ca. 30%) in favor of fish. Because the overall lipid content calculated for the freshwater fish and game source is low, and the protein content high, if the prior is engaged, the model compensates for these low energy sources by increasing the carbohydrate-rich plant portion. The health risks associated with high protein diets in areas with low plant productivity and low lipid availability – in its symptomatic form often referred to as ‘protein poisoning’ or ‘rabbit starvation’– is a phenomenon known to the people of the boreal and subarctic regions [114], and both subsistence strategies that temporally exceed the ca. 45% physiological protein limit, as well as adaptive strategies to prevent this are common. Game and freshwater fish are often lean, but it is possible to target the fat that is present, a strategy pursued ethnographically by Cree hunters in a similar boreal environment to that of Karelia. This includes bone marrow and beaver tails, and an often high appreciation for these fatty parts [108]. If fat is included as a distinct food source in the FRUITS models, it too significantly affects the modelled dietary contributions from plants, fish and game and overall lowers the plant contribution (see SI2, Fig S9 in S1 File). Accordingly, one possible explanation for the discrepancy between the modelled results and ethnographic records could be a systematic underestimation of the lipid contribution to the diet. The lack of faunal preservation in the wider Onega area has so far not allowed the identification of specific fat acquiring strategies based on bone finds and modifications (e.g., high fragmentation typically associated with the extraction of bone grease [225]). The high prevalence of terrestrial fauna at YOO, the bone pendants produced from split long bones, which could be related to marrow extraction, and the tail bones of a beaver recovered from one of the burials (burial 100) [52] (identification by K. Mannermaa), could be interpreted with their value as a fat resource in mind.

With the limited background data both in terms of isotopes and archaeological assemblages currently accessible to us, it is not possible to conclusively accept or reject the results of the dietary mixing models. Yet, while we do consider it possible that the plant dietary portion exceeded the often referenced 25% for boreal HGF, we would argue that the models likely underestimate the contribution of terrestrial game and fats to the YOO diet. A more realistic estimate might be the 30–40% plant diet suggested by Zvelebil for the European Mesolithic under consideration of lean freshwater fish, game and human physiological needs [209]. Bearing in mind the similarity of our models’ proposed estimates to other previous BMM assessments for archeological north-eastern European inland HGF sites, a more systematic re-evaluation of the relationship between modelling results and ethnographic records and their implications should be conducted in the future.

Nevertheless, even with this conundrum maintained, all other evidence points to high-trophic-level aquatic resources as the key component of YOO diet, and an overall high-protein diet. This is attested by what seems to be a high diet-to-collagen fractionation in the human *δ*^15^N values, which is linked to high protein content, with excessive (^15^N-depleted) nitrogen being shed through enhanced urea excretion leading to ^15^N-enriched tissue values [226–228]. The above supports applying a high TEF value (i.e., variant a, TEF = 5.5‰) for nitrogen in the dietary mixing model as the most appropriate choice. Similarly, while the connections between dietary changes and changes in individual amino acid isotope values are still poorly understood, experimental studies using animal models have linked varying *δ*^13^C_Glx_ values with changes in macronutrient composition [229]. It has also been suggested that high *δ*^13^C_Gly_ values may be connected to excess protein consumption and its synthesis into lipids, energy and glycine [23,36,40,230]. Changes in both proxies have further been linked to an overall imbalance in the ratio of protein to energy [22,231]. Accordingly, the extremely low *δ*^13^C_Glx_ and relatively high *δ*^13^C_Gly_ values we observed for the YOO individuals could tentatively be interpreted as being the result of a very high contribution of protein to the diet.

### One site, one time, one diet?

**Fig 7:**
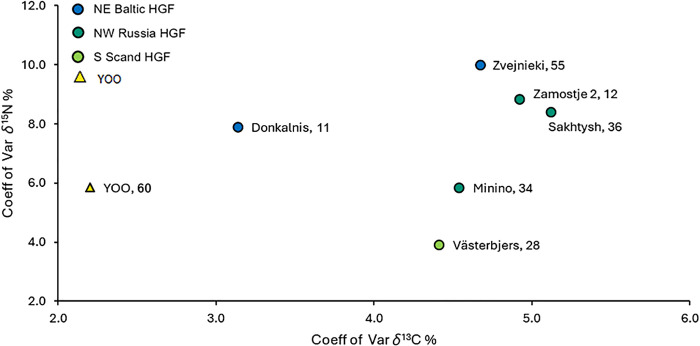
Coefficient of variation for δ^13^C and δ^15^N values measured from HGF individuals from circum-Baltic sites. Only sites with more than ten individuals analyzed are included (numbers refer to sample size). For Zvejnieki only HGF individuals are shown. For summary statistics see SI3, Table S7 in S1 Data.

One repeated observation for all the presented isotope proxies, which also translated into modelling results, is the relatively narrow range of values observed for the YOO humans. This implies limited variability in the diets of individuals, at least of isotopically distinct foods. While comparing isotopic variation between site is difficult both due to differences in baselines and high variation in numbers of recovered individuals, the values we observed at YOO showed intra-site consistency with a standard deviation of only 0.4‰ in *δ*^13^C_col_ and 0.9‰ in *δ*^15^N_col_ and coefficients of variation of 2.1% and 5.9% respectively (the two outlier values are retained to facilitate comparison with other sites, where outliers were similarly not removed; without the outlier SD for *δ*^15^N_col_ is 0.8‰ and *δ*^13^C_col_ 0.4‰). This is lower than seen in the nearest HGF sites with more than ten available measurements, even though, with the exception of Västerbjers on Gotland, Sweden, all are inland sites with a strong freshwater component (Fig 7). This low variation at YOO is surprising, as with 60 individuals the overall sample size is high and HGFs commonly tend to exhibit a higher baseline variability in dietary isotope values, compared to agropastoral societies, due to their generally more diverse resource base and the impact of mobility and seasonality. In the case of YOO though, the distribution forms a narrow cluster, whereas other sites in the wider region show a broader spread of values even with substantially smaller sample sizes.

In general, this likely indicates that the diet consumed by the people buried at YOO was overall very isotopically similar and there were no obvious subgroups that persistently engaged in different subsistence practices as observable in their isotope values. A potential reduction in the resource spectrum associated with the 8.2 ka event, driven by its colder and drier climate, could have contributed to this result. However, any direct impact on human diet due to the climatic downturn remains uncertain owing to a lack of data for human remains from pre- and post-8.2 ka event sites around the lake, and no evidence for related changes in material culture has been found so far [232]. Cultural practices, such as a high degree of food sharing, which may have been more strongly enacted in response to deteriorating environmental conditions, could similarly lead to reduced variability in isotope values. However, the most important factor influencing the observed homogeneity is likely the narrow temporal window of one to three centuries during which the cemetery was in use [1]. Most other northern HGF cemeteries were used over substantially longer periods compared to YOO, e.g., over a millennium at Zvejnieki, Zamostje and Minino [2,63,153]. Environmental changes affecting the local isoscape – e.g., changes in the distribution of the fauna, lake nitrogen cycle, or in subsistence practices over time – would have been substantially less likely to be impactful during the 100–300 years of activity at YOO.

The only exceptions to the observed homogeneity at the site are the two observed outliers: burial 59, with slightly lower *δ*^13^C_col_ values, and burial 57, with substantially lower *δ*^15^N_col_ values. Both cases were already identified as isotopically unusual previously [1] and belong to the most richly furnished burials at the site. In the case of burial 59 it needs to be noted that the measurements – while still done separately – were associated with the radiocarbon analysis as there was not enough bone collagen remaining for another run with appropriate calibration standards [1]. Considering the higher uncertainty of these measurements and the overall narrow range of *δ*^13^C_col_ values at the site, there is a chance that the divergence between the values of 59 (−21.5‰) and the site mean (−20.0 ± 0.4), could be statistically significant but not interpretable in dietary or physiological terms. However, Schulting et al. [1] did also observe variation in freshwater reservoir effects between individuals and suggested they could be a result of acquiring fish from different parts of the large lake and its surrounding rivers. This could also explain the lower values of this individual. Support for this hypothesis may come from the individuals’ strontium isotope values, which fall within the upper tail of the YOO distribution [66], possibly indicating a slightly divergent background from the majority of burials.

The *δ*^15^N_col_ measurement for burial 57 (12.5‰ vs. the site mean of 16.1 ± 0.9) indicate their adult diet either accessed fish with very different *δ*^15^N values than everyone else or, more likely, had a considerably more terrestrial (game) focused dietary intake than any other individual, ingesting ca. 50% less aquatic protein than the YOO average. The radiocarbon analysis of this burial showed no ^14^C offset compared to an analyzed elk tooth pendant from the same grave, supporting little impact from freshwater reservoirs on the carbon isotope signal [1]. Considering that this individual’s mid-childhood isotope values (*δ*^13^C = −20.1‰ and *δ*^15^N‰ = 16.6‰, Eckelmann et al. in prep.) conform to the YOO norm and their strontium isotope values similarly point to the areas commonly accessed by the YOO community in the north of Lake Onega [66], this could either indicate a dietary choice or time spent outside of the shared YOO subsistence culture in the decade before the individual’s death. This burial is part of triple grave 55–57, one of the most richly furnished at the site [52], which also contained one of two identified individuals with non-local strontium values, further supporting the unique status of the individuals in this grave.

## Conclusions

Within the archeological hunter-gatherer-fisher groups of the circum-Baltic, YOO forms a unique cluster distinguished by *δ*^15^N values high enough to be comparable to Scandinavian coastal groups, combined with *δ*^13^C values at the higher end of typical C3 terrestrial/freshwater environments. Assessing the various complementary isotope proxies, we argue that the distinct YOO isotopic signature was not derived from marine resource consumption and also reject the hypothesis of potential freshwater seal hunting at Lake Onega. Instead, we propose that the high human *δ*^15^N values could have been acquired from resources within the Lake Onega system, with a high protein intake leading to a large diet-to-tissue offset. Overall, the multi-isotopic proxies confirm the dominance of protein from aquatic sources, in line with previous assessments. Attempts at modelling dietary proportions via the Bayesian mixing models FRUITS returned quantities of plant, game and fish consumption comparable to other inland HGF sites in north-eastern Europe, with surprisingly low contributions of terrestrial game and a surprisingly high importance of plant foods. Both findings contradict ethnographic data on boreal HGF subsistence, and we propose that the model may underestimate the consumption of animal-derived lipids, recommending further investigation of the discrepancy between archaeological dietary isotope mixing models and the ethnographic record. Lastly, we confirm that the inter-individual variation in isotopic values at YOO is very low in all measured proxies and no significant differences between individuals interred in different areas of the burial site or between sexes are observed. This relative homogeneity, compared with other sites, is likely a result of the cemetery’s short duration of use, reflecting a specific set of cultural and economic practices, although the presence of two outliers also hints at diet choice or individual mobility. Other than the site’s short period of use, a relative lack of mobility between YOO and other groups, as has been previously proposed, and common HGF practices of sustained within-group food sharing, could have contributed to the observed homogeneity. In the future more detailed observations of the dietary baseline at Lake Onega and its surrounding during, before and after the usage of the YOO burial site, other methods, e.g., dental calculus analyses, and a more extensive study of Lake Onega’s paleo-ecosystem will provide further insights into past diets and lifeways at YOO.

## Supporting information

S1 File SI1–2 – Extended laboratory methods, results and figures. (DOCX)

S1 Data SI3 – Data. (XLSX)
