# Supplementary material for: Investigating human paleodiet at Mesolithic Yuzhniy Oleniy Ostrov, Karelia using a multi-proxy stable isotope approach
Source: PLoS One. 2026 Jan 7;21(1):e0338887. doi: 10.1371/journal.pone.0338887 (PMC12779038; doi:10.1371/journal.pone.0338887)
Supplement: S1 File — (DOCX) [file pone.0338887.s001.docx]

**Investigating human paleodiet at Mesolithic Yuzhniy Oleniy Ostrov, Karelia using a multi-proxy stable isotope approach**

**Supplementary Information 1-2**

Rebekka Eckelmann^1*^, Laura Arppe^2^, Rick J. Schulting^3^, Sambit Ghosh^4^, Jakub Trubač^5^, Aneta Kuchařová^5^ Matthew J. Wooller^4,6^, Dmitry Gerasimov^7^, Vyacheslav Moiseyev^7^, Kristiina Mannermaa^1^

1 Department of Cultures, University of Helsinki, Helsinki, Finland

2 Finnish Museum of Natural History, University of Helsinki, Helsinki, Finland

3 School of Archaeology, University of Oxford, Oxford, UK

4 Alaska Stable Isotope Facility, Institute of Northern Engineering, University of Alaska Fairbanks, Fairbanks, USA

5 Institute of Geochemistry, Mineralogy and Mineral Resources, Faculty of Science, Charles University, Prague, Czech Republic

6 College of Fisheries and Ocean Sciences, University of Alaska Fairbanks, Fairbanks, USA

7 Peter the Great Museum of Anthropology and Ethnography (Kunstkamera), Russian Academy of Sciences, St Petersburg, Russia

*Corresponding author

E-mail: [rebekka.eckelmann@helsinki.fi](mailto:rebekka.eckelmann@helsinki.fi)

# **SI 1. Extended laboratory analysis**

## ***δ*^13^C_col_ and *δ*^15^N_col_**

Collagen samples analyzed for human *δ*^13^C and *δ*^15^N in the course of this study were prepared and measured at the Laboratory of Chronology, Finnish Museum of Natural History, Helsinki as part of a study focused on sequential dentine *δ*^13^C and *δ*^15^N analysis.

Collagen extraction was conducted following the procedure described for the preparation of sequential samples in Czermak et al. [1], which included the demineralization of a 2 mm thick crown to root apex cross-cut of the sampled tooth in 0.5 M HCl at 4°C until fully demineralized, removal of humic acids through a soak in 0.1 M NaOH followed by a rinse in 0.5 M HCl and subsequent rinsing in deionized double-distilled water (DDH_2_O) to neutrality. Afterwards the demineralized tooth slice was sampled sequentially with a 1 mm KAI Medical biopsy punch. Samples were then freeze-dried for 8 h. The modern fish and archaeological faunal samples analyzed here were processed through the same procedure, except that chunks were cut from tooth dentine and fish bone respectively without sequential sampling.

Subsequently, approximately 250 μg per sample were then weighed into tin capsules and measured for *δ*^13^C and *δ*^15^N ratios using an EA-IRMS (Elemental Analyzer-IRMS; NC 2500 + Thermo Fisher ScientificDelta V Advantage). Samples were calibrated using the internationally recognized standards USGS-40 and USGS-41 and quality was monitored with in-house standards of caffeine powder, elk, ibex and camel bone collagen (Table S1). As an additional measure to ensure comparability, internal standards (COW and SEAL collagen, Table S1) previously used at the Oxford stable isotope laboratory to normalize collagen samples from YOO were included in Helsinki measurements.

**Table S1: Measured and calibrated values of all reference materials included in the runs.** Except for USGS40 and USGS41 “True” refers to the expected value according to long-term averages. The USGS references were used for data normalisation.

|  | *δ*^13^C | | | | | *δ*^15^N | | | | |  |
| --- | --- | --- | --- | --- | --- | --- | --- | --- | --- | --- | --- |
|  | Measured | SD | Calibrated | SD | **True** | Measured | SD | Calibrated | SD | **True** | n |
| USGS40 | -26.3 | 0.1 | -26.4 | 0.1 | **-26.4** | -4.6 | 0.1 | -4.5 | 0.1 | **-4.5** | **77** |
| USGS41 | 36.7 | 0.2 | 37.8 | 0.4 | **37.6** | 46.3 | 0.5 | 47.4 | 0.6 | **47.6** | **63** |
| Caffeine | -37.8 | 0.1 | -38.1 | 0.1 | **-38.1** | -7.6 | 0.1 | -7.7 | 0.2 | **-7.6** | **68** |
| Seal | -12.8 | 0.2 | -12.7 | 0.2 | **-12.5** | 15.5 | 0.2 | 16.1 | 0.3 | **16.1** | **36** |
| Cow | -24.5 | 0.1 | -24.6 | 0.1 | **-24.3** | 7.6 | 0.1 | 8.1 | 0.1 | **7.9** | **26** |
| Ibex | -24.3 | 0.1 | -24.5 | 0.1 | **-24.7** | 6.7 | 0.1 | 6.9 | 0.1 | **7.2** | **6** |
| Camel | -14.9 | 0.2 | -14.7 | 0.3 | **-14.8** | 7.6 | 0.4 | 8.1 | 0.2 | **8.2** | **16** |
| Elk | -24.1 | 0.3 | -24.1 | 0.3 | **-24.1** | 2.4 | 0.4 | 2.7 | 0.2 | **2.8** | **15** |

## ***δ*^13^Capa**

To prepare for the analysis of *δ*^13^C on structural carbonate from tooth enamel bioapatite, the tooth was mechanically cleaned to remove surface contaminants and then ultrasonically cleaned in deionized DDH_2_O for 20 min. One sample contained ca. 2-4 mg of enamel powder drilled via a handheld Proxxon device. The samples were loaded into microcentrifuge tubes and left to rest in a 2.5 % NaOCl solution for 17h at room temperature. Afterwards samples were rinsed five times with DDH_2_O and then soaked in a 1 M Ca acetate+CH3COOH buffer solution for 30 minutes, rinsed again and then dried freeze-dried.

*δ*^13^C measurements were undertaken at the Faculty of Science, Charles University, Prague, as a conventional paired analysis of carbon and oxygen stable isotopes employing a *GasBench II* (ThermoFisher Scientific) equipped with a *CTC Combi-Pal* (PALSYSTEM) autosampler and linked to a *MAT253* isotope ratio mass spectrometer (ThermoFisher Scientific) in a *Continuous Flow IV* (ThermoFisher Scientific) system. Analysis of *δ*^18^O was undertaken simultaneously but not included in this study.

About 500-550 µg of enamel powder was weighed into 10-ml round-bottomed borosilicate exetainers and sealed butyl rubber septa in blue caps (Labco Limited; United Kingdom). The exetainers were placed into aluminium trays kept at 72.0 ± 0.1°C. During measuring the exetainers were automatically flushed with 5.0 purity He by penetrating the septa using a double-hole needle at a flow rate of 120 mL/min. Afterwards, 15-17 drops of phosphoric acid (density 1.91 g/cm^3^) were deposited in each exetainer manually using a syringe.

Following water removal using Nafion traps, CO2 was separated from other components using a gas chromatographic column (Poraplot Q with fused-silica tubing, 25m×0.32mm) heated to 70.0°C, and the peak corresponding to this CO_2_ is then passed via an open split into the mass spectrometer. Each sample run started out with peak centering, followed by four rectangular-shaped peaks of CO_2_ reference gas, after which eight successive sample peaks are produced by delivering sequential aliquots of pure CO_2_ into the ion source of MS. The internal precision (SD) measured over sample peaks is typically 0.02 ‰ and 0.09 ‰ for raw *δ*^13^C and *δ*^18^O values, respectively. Calibration of the raw results versus the V-PDB scale were achieved using international reference materials NBS-18 and IAEA-603 (International Atomic Energy Agency, Vienna, Austria).

## **CSIA-AAs**

4-6 mg of each collagen sample was processed for measuring *δ*^15^N and *δ*^13^C ratios of amino acids (AAs) at the Alaska Stable Isotope Facility (ASIF), University of Alaska Fairbanks (UAF) following published literature [2,3]. Briefly, collagen samples were hydrolyzed using 6 N HCl at 110 °C for 20 h and hydrolysate was lipid extracted with 6:5 (v/v) hexane and dichloromethane [2,4]. An internal norleucine standard was added to the remaining aqueous phase that contains AAs and was then dried under N_2_ flow. The dried samples were derivatized to volatile AA n-acetyl-isopropyl (NAIP) esters via propylation and acetylation [2,5]. 1 µl AA-derivatives were injected into a Thermo Trace GC 1310 with a splitless injector held at 240 °C with a constant He flow of 1.4 ml/min for determining *δ*^15^N values of AAs. The injector was at 280 °C with a programable He flow: 1ml/min (0.5 min hold) to 1.5ml/min at 1ml/min (19 min hold), and then 2.2ml/min at 1ml/min (11 min hold) for determining *δ*^13^C values of AAs.

For determining *δ*^15^N values, AA molecules were separated using Agilent DB-35 GC column (60m × 0.32mm × 0.50µm) with the following GC oven temperature program: 40 °C (5 min hold) ramped to 120 °C at 15 °C/min, then to 180 °C at 4 °C/min to 210 °C at 1.5°C/min, and finally to 280°C at 6 °C/min (8 min hold). For determining *δ*^13^C values, AA molecules were separated using Agilent VF-35ms GC column (30m × 0.32mm × 1µm) with the following GC oven temperature program: 80 °C (1 min hold) ramped to 135 °C at 20 °C/min, to 160 °C at 5 °C/min (3 min hold), and then to 300 °C at 8 °C/min (3 min hold). Representative chromatograms illustrating the separation of measured peaks are provided in SI2, Fig. S1. The GC effluent was routed through the Isolink II interference with a combustion oven at 1000 °C and the evolved N_2_ and CO_2_ gases were introduced to Thermo Delta V^Plus^ isotope ratio mass spectrometer via Conflo IV. External AA standard mix UAF AA1 and UAF AA2 were derivatized and ran with the samples to report the *δ*^15^N and *δ*^13^C values of AAs with respect to their respective international standards.

The slope and intercept of measured and known *δ*^15^N values of AAs in external UAF AA1 and AA2 standards were used to correct the measured *δ*^15^N_AAs_ in samples. The average slope and intercept were 1 and 0.1, respectively in the external standard mix. The difference between measured and known *δ*^15^N values of internal standard norleucine and external standard caffeine were 1.1‰ and 0.4‰, respectively. Typically, instrument precision for measuring d^15^N values of molecular organic compounds is <0.5 ‰.

The derivatization chemicals add carbon to the derivatives, and therefore measured *δ*^13^C values of AAs in samples were corrected against known values of AA standards that were derivatized with each batch of samples (e.g., Johnson et al., 2021). The final *δ*^13^C value for each AA in a sample was determined as follows:

$$\delta^{13}C_{{AA}_{Sample}}=\frac{\delta^{13}C_{{AA}_{dSA}}-\delta^{13}C_{{AA}_{dST}}+\delta^{13}C_{{AA}_{ST}}\times P_{ST}}{P_{ST}}$$

where dSA and dST refer to the derivatized sample and AA standard, respectively, and P*_ST_* represents the mole fraction of carbon in the derivatized AAs from the un-derivatized AAs. $\delta^{13}C_{\mathrm{AA}_{\mathrm{ST}}}$ is the known value of the AA standard. Specifically, we corrected UAF AA2 using UAF AA1 and the corrected and known/true values of AAs in UAF AA2 corelated well with R^2^=0.99, slope=0.98 and intercept=0.01. The difference between measured and known *δ*^13^C values of internal standard norleucine was 0.1‰. Typically, instrument precision for measuring *δ*^13^C values in molecular organic compound is <0.2‰.

The relationship between proline and hydroxyproline was monitored as another quality indicator with both showing high correlation and regression lines close to the 1:1 line (*δ*^15^N_Hyp vs Pro_: R^2^ = 0.998, slope = 1.01 and *δ*^13^C_Hyp vs Pro_: R^2^ = 0.996, slope = 1.01), indicating good data quality (SI2, Fig. S2) [6].

# **SI 2. Extended results and figures**

**a**


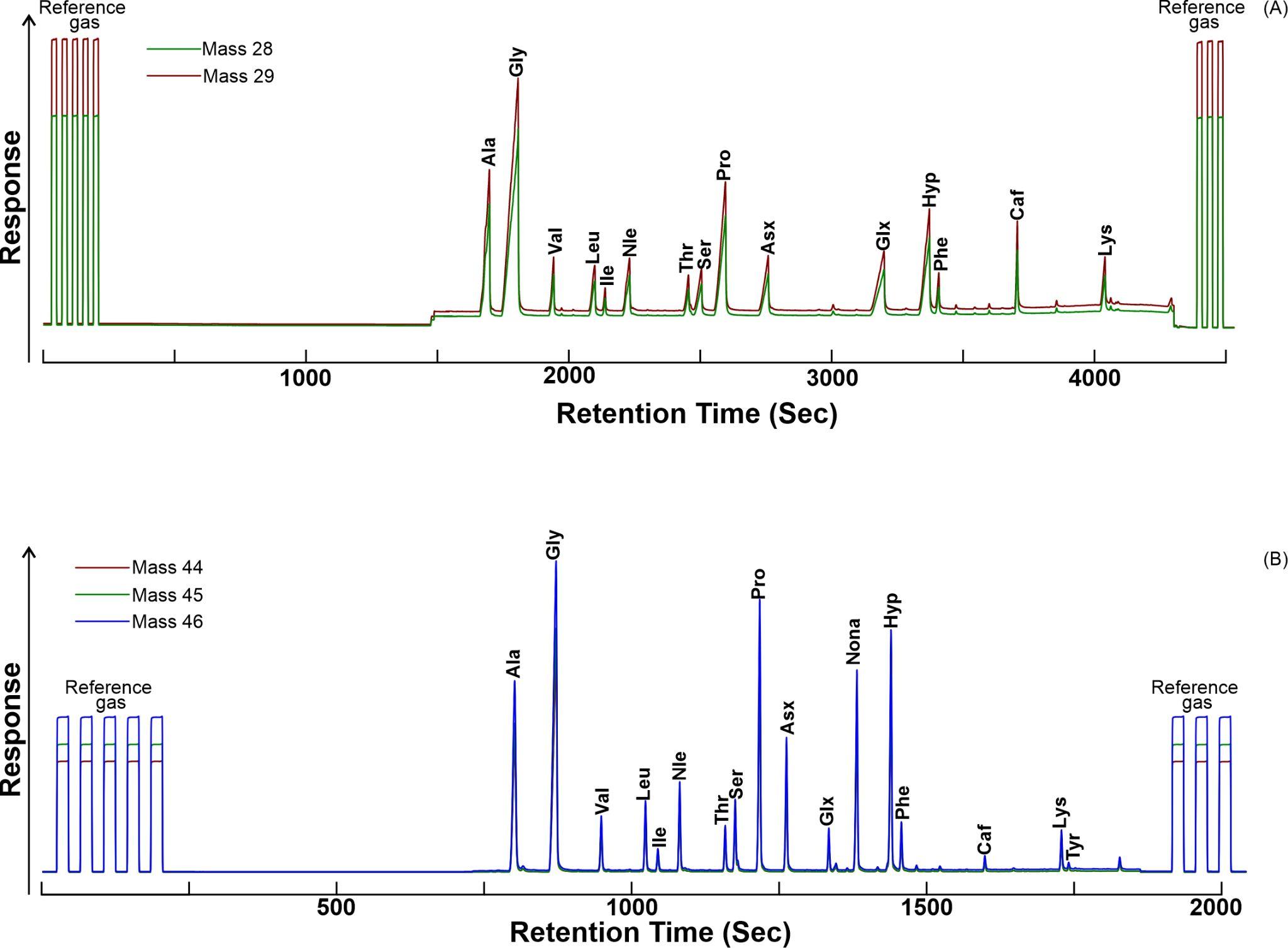


**b**

**Fig. S1:** Representative chromatograms for sample 56/KK7 show different amino acids (AA) measured for (a) nitrogen and (b) carbon isotopes. Both chromatograms indicate a good baseline separation for the reported nitrogen and carbon isotope values of AAs. Reference gas, AAs (Ala: Alanine, Gly: Glycine, Val: Valine, Leu: Leucine, Ile: Isoleucine, Thr: Threonine, Ser: Serine, Pro: Proline, Asx: Aspartic acid, Glx: Glutamic acid, Hyp: Hydroxyproline, Phe: Phenylalanine, Lys: Lysine, Tyr: Tyrosine); internal AA standard (Nle: Norleucine), external standards (Nona: Nonadecane and Caf: Caffeine) are annotated.

**
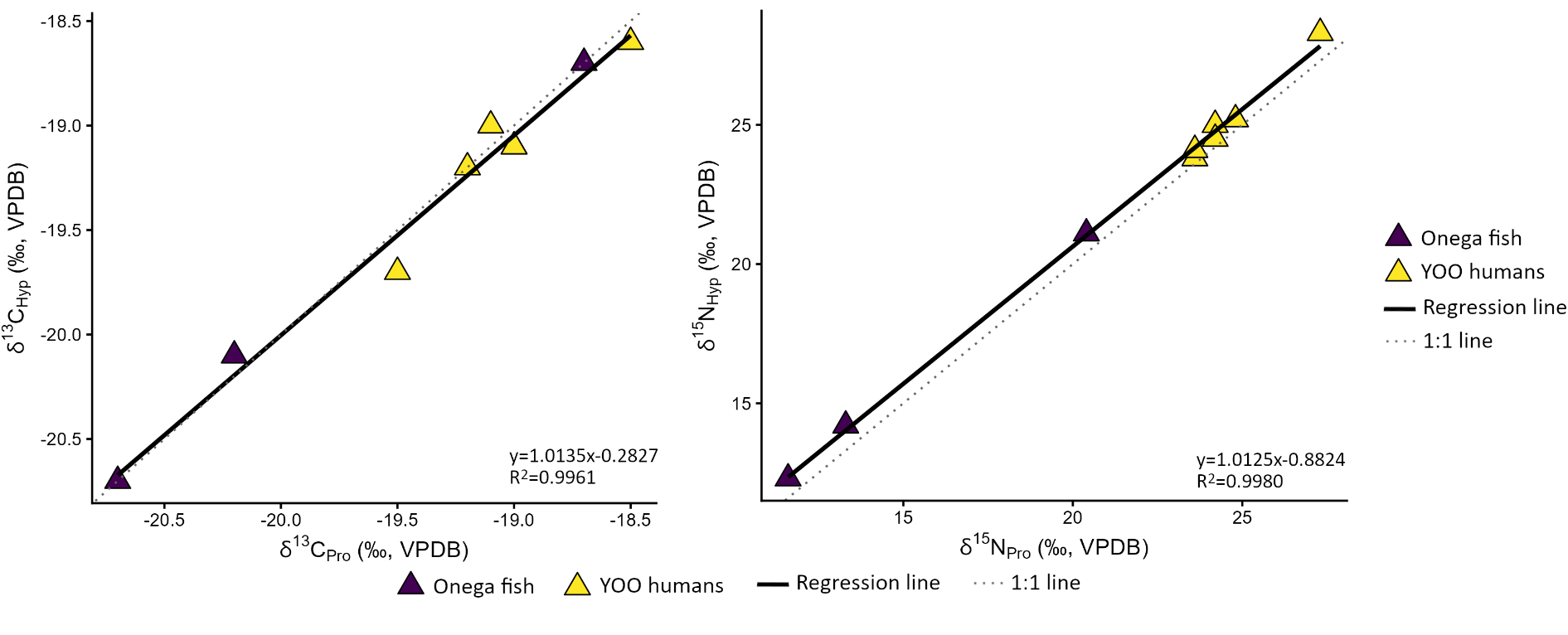
Fig. S2:** Quality control plot showing the relationship between proline (Pro) and hydroxyproline of the studied collagen samples for *δ*^13^C (a) and *δ*^15^N (b).

**b**

**a**

**
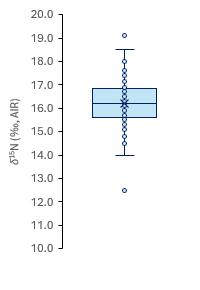

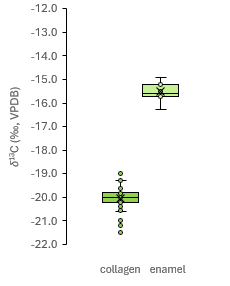
Fig S3:** Distribution of *δ*^13^C values measured on human collagen and enamel apaptite (a), as well as as *δ*^15^N measured on human bulk collagen (b). Statistical outliers are marked with asterisk. Collagen values derive partially from [7,8].

*

*

**b**

**a**


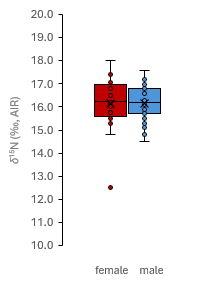

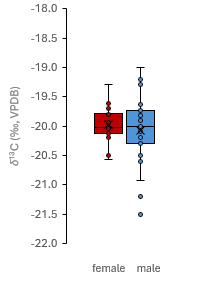


**Fig S4:** *δ*^13^C (a) and *δ*^15^N (b) values measured on human collagen separated by sex (male = 28, female = 25) (after Batanina et al. in prep.)

**a**

**b**

*

*


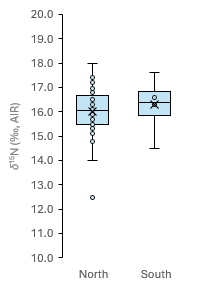

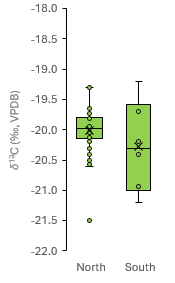


**b**

**a**

**Fig S5:** *δ*^13^C (a) and *δ*^15^N (b) values measured on human collagen separated for the northern (n = 51) and southern (n = 7) section of the YOO burial site.

*

*


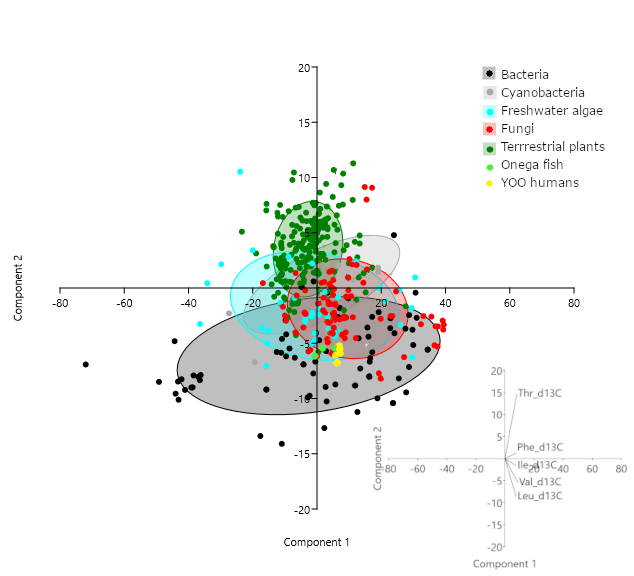


**Fig S6:** Plotted results of component 1 (86.6%) and 2 (7.9%) of the principal component analysis including normalized *δ*^13^C values of isoleucine, leucine, phenoalanine, threonine and valine (ellipses at 65%), which did not separate between bacteria, cyanobacteria, freshwater algae and fungi to a degree sufficient for further use in dietary mixing models. Data for basal producers from [9–28].


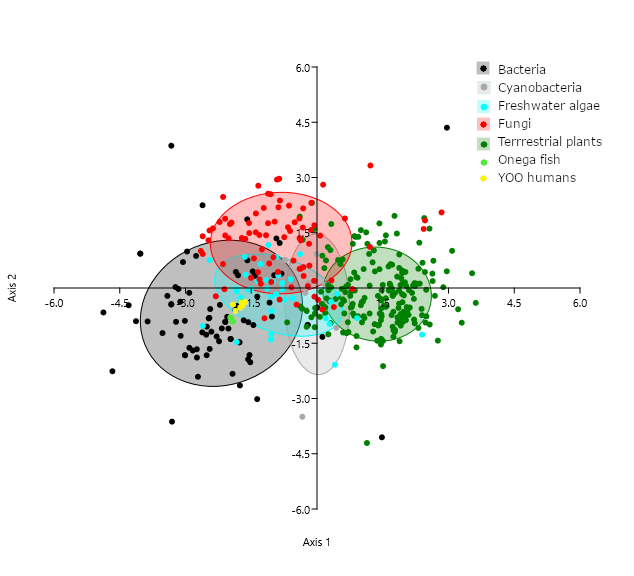


**Fig S7:** Plotted results of the linear discriminant analysis and confusion matrix for normalized *δ*^13^C values of isoleucine, leucine, phenoalanine, threonine and valine (ellipses at 65%). With 80.4% and 16.1% percentages of discrimination in the first and second axis, it did not separate between bacteria, cyanobacteria, freshwater algae and fungi to a degree sufficient for further use in dietary mixing models. Source data for basal producers referred in Fig. S4 above.


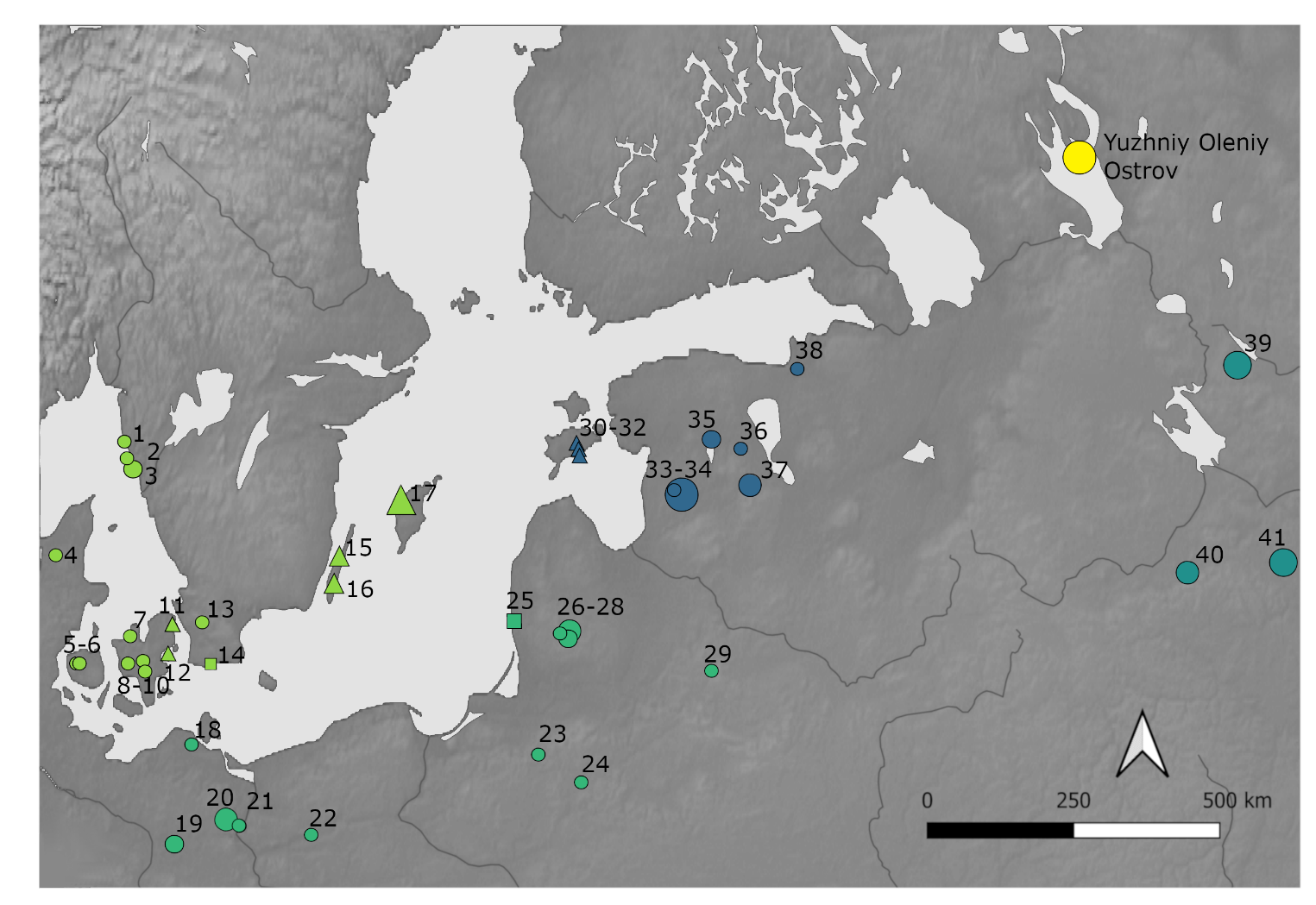


**Fig S8:** Detail of Fig 4 with numbers for included sites. Sites are as follows: 1. Uleberg, 2. Evensås, 3. Huseby Klev, 4. Hedegård, 5. Ageröd, 6. Koelbjerg, 7. Mullerup, 8. Kongemose, 9. Tømmerupsgård Mose, 10. Holmegård, 11. Nivågård, 12. Køge Sønakke, 13. Hanaskede, 14. Skateholm, 15. Köpingsvik, 16. Alby, 17. Västerbjers, 18. Steinhagen, 19. Friesack, 20. Groß Fredenwalde, 21. Criewen, 22. Krzyż Wielkopolski, 23. Pierkunowo-Giżycko, 24. Woźna Wieś, 25. Šventoji, 26. Daktariškė, 27. Donkalnis, 28. Spiginas, 29. Kretuonas, 30. Kõljala, 31. Kõnnu, 32. Nakamäe, 33. Riņņukalns, 34. Zvejnieki, 35. Kivisaare, 36. Veibri, 37. Tamula, 38. Narva Joaorg, 39. Minino, 40. Zamostje, 41. Sakhtysh. The site map was created using Natural Earth public domain data.


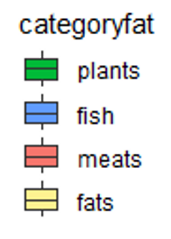

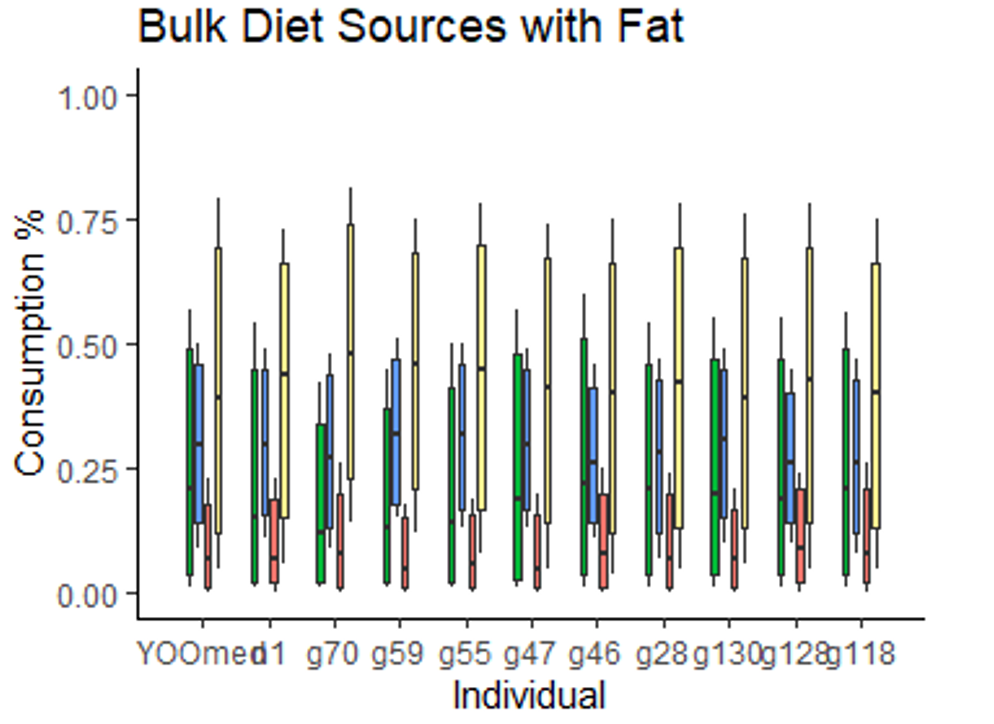


**Fig S9:** Results for bulk dietary model 1.2a (based on *δ*^13^C_col_, *δ*^13^C_apa_ and *δ*^15^N_col_ , a wider local baseline and applying a TEF for *δ*^15^N of 5.5 ± 0.5‰) inlcuding (game derived) fat as a distinct fourth dietary source in addition to fish, game and plants. Source values for fat are set as *δ*^13^C_col_energy_ = -29.5 ± 1.4‰ and *δ*^13^C_apa_energy_ = -25.6 ± 1.4‰. In this model it is assumed that the game (meat) portion only contributes protein and the (game) fat protion only contributes energy.

# References:

1. Czermak A, Fernández‐crespo T, Ditchfield PW, Lee‐thorp JA. A guide for an anatomically sensitive dentine microsampling and age‐alignment approach for human teeth isotopic sequences. Am J Phys Anthropol. 2020;173: 776. doi:10.1002/ajpa.24126

2. Trifari MP, Wooller MJ, Rea L, O’Hara TM, Lescord GL, Parnell AC, et al. Compound-specific stable isotopes of amino acids reveal influences of trophic level and primary production sources on mercury concentrations in fishes from the Aleutian Islands, Alaska. Science of The Total Environment. 2024;908: 168242. doi:10.1016/J.SCITOTENV.2023.168242

3. Barst BD, Wooller MJ, O’Brien DM, Santa-Rios A, Basu N, Köck G, et al. Dried Blood Spot Sampling of Landlocked Arctic Char (Salvelinus alpinus) for Estimating Mercury Exposure and Stable Carbon Isotope Fingerprinting of Essential Amino Acids. Environ Toxicol Chem. 2020;39: 893–903. doi:10.1002/ETC.4686

4. Johnson JJ, Shaw PA, Oh EJ, Wooller MJ, Merriman S, Yun HY, et al. The carbon isotope ratios of nonessential amino acids identify sugar-sweetened beverage (SSB) consumers in a 12-wk inpatient feeding study of 32 men with varying SSB and meat exposures. Am J Clin Nutr. 2021;113: 1256–1264. doi:10.1093/AJCN/NQAA374

5. Styring AK, Fraser RA, Arbogast RM, Halstead P, Isaakidou V, Pearson JA, et al. Refining human palaeodietary reconstruction using amino acid *δ*15N values of plants, animals and humans. J Archaeol Sci. 2015;53: 504–515. doi:10.1016/J.JAS.2014.11.009

6. O’Connell TC, Collins MJ. Comment on “Ecological niche of Neanderthals from Spy Cave revealed by nitrogen isotopes of individual amino acids in collagen” [J. Hum. Evol. 93 (2016) 82–90]. J Hum Evol. 2018;117: 53–55. doi:10.1016/J.JHEVOL.2017.05.006

7. Schulting RJ, Mannermaa K, Tarasov PE, Higham T, Ramsey CB, Khartanovich V, et al. Radiocarbon dating from Yuzhniy Oleniy Ostrov cemetery reveals complex human responses to socio-ecological stress during the 8.2 ka cooling event. Nature Ecology & Evolution 2022 6:2. 2022;6: 155–162. doi:10.1038/s41559-021-01628-4

8. Wood RE, Higham TFG, Buzilhova A, Suvorov A, Heinemeier J, Olsen J. Freshwater Radiocarbon Reservoir Effects at the Burial Ground of Minino, Northwest Russia. Radiocarbon. 2013;55: 163–177. doi:10.2458/azu_js_rc.v55i1.16448

9. Arsenault ER, Liew JH, Hopkins JR. Substrate composition influences amino acid carbon isotope profiles of fungi: implications for tracing fungal contributions to food webs. Environ Microbiol. 2022;24: 2089–2097. doi:10.1111/1462-2920.15961

10. Besser AC, Elliott Smith EA, Newsome SD. Assessing the potential of amino acid *δ*13C and *δ*15N analysis in terrestrial and freshwater ecosystems. Journal of Ecology. 2022;110: 935–950. doi:10.1111/1365-2745.13853

11. Elliott Smith EA, Fox MD, Fogel ML, Newsome SD. Amino acid *δ*13C fingerprints of nearshore marine autotrophs are consistent across broad spatiotemporal scales: An intercontinental isotopic dataset and likely biochemical drivers. Funct Ecol. 2022;36: 1191–1203. doi:10.1111/1365-2435.14017

12. Fogel ML, Tuross N. Extending the limits of paleodietary studies of humans with compound specific carbon isotope analysis of amino acids. J Archaeol Sci. 2003;30: 535–545. doi:10.1016/S0305-4403(02)00199-1

13. Gómez C, Larsen T, Popp B, Hobson KA, Cadena CD. Assessing seasonal changes in animal diets with stable-isotope analysis of amino acids: a migratory boreal songbird switches diet over its annual cycle. Oecologia. 2018;187: 1–13. doi:10.1007/S00442-018-4113-7

14. Jarman CL, Larsen T, Hunt T, Lipo C, Solsvik R, Wallsgrove N, et al. Diet of the prehistoric population of Rapa Nui (Easter Island, Chile) shows environmental adaptation and resilience. Am J Phys Anthropol. 2017;164: 343–361. doi:10.1002/AJPA.23273

15. Larsen T, Taylor DL, Leigh MB, O’Brien DM. Stable isotope fingerprinting: a novel method for identifying plant, fungal, or bacterial origins of amino acids. Ecology. 2009;90: 3526–3535. doi:10.1890/08-1695.1

16. Larsen T, Wooller MJ, Fogel ML, O’brien DM. Can amino acid carbon isotope ratios distinguish primary producers in a mangrove ecosystem? 2012. doi:10.1002/rcm.6259

17. Larsen T, Ventura M, Andersen N, O’Brien DM, Piatkowski U, McCarthy MD. Tracing Carbon Sources through Aquatic and Terrestrial Food Webs Using Amino Acid Stable Isotope Fingerprinting. PLoS One. 2013;8: e73441. doi:10.1371/JOURNAL.PONE.0073441

18. Larsen T, Pollierer MM, Holmstrup M, D’Annibale A, Maraldo K, Andersen N, et al. Substantial nutritional contribution of bacterial amino acids to earthworms and enchytraeids: A case study from organic grasslands. Soil Biol Biochem. 2016;99: 21–27. doi:10.1016/J.SOILBIO.2016.03.018

19. McCarthy MD, Lehman J, Kudela R. Compound-specific amino acid *δ*15N patterns in marine algae: Tracer potential for cyanobacterial vs. eukaryotic organic nitrogen sources in the ocean. Geochim Cosmochim Acta. 2013;103: 104–120. doi:10.1016/j.gca.2012.10.037

20. Liew JH, Chua KWJ, Arsenault ER, Thorp JH, Suvarnaraksha A, Amirrudin A, et al. Quantifying terrestrial carbon in freshwater food webs using amino acid isotope analysis: Case study with an endemic cavefish. Methods Ecol Evol. 2019;10: 1594–1605. doi:10.1111/2041-210X.13230

21. Manlick PJ, Newsome SD. Stable isotope fingerprinting traces essential amino acid assimilation and multichannel feeding in a vertebrate consumer. Methods Ecol Evol. 2022;13: 1819–1830. doi:10.1111/2041-210X.13903

22. Manlick PJ, Cook JA, Newsome SD. The coupling of green and brown food webs regulates trophic position in a montane mammal guild. Ecology. 2023;104. doi:10.1002/ECY.3949

23. Paolini M, Ziller L, Laursen KH, Husted S, Camin F. Compound-Specific *δ*15N and *δ*13C Analyses of Amino Acids for Potential Discrimination between Organically and Conventionally Grown Wheat. J Agric Food Chem. 2015;63: 5841–5850. doi:10.1021/ACS.JAFC.5B00662/SUPPL_FILE/JF5B00662_SI_001.PDF

24. Pollierer MM, Scheu S, Tiunov A V. Isotope analyses of amino acids in fungi and fungal feeding Diptera larvae allow differentiating ectomycorrhizal and saprotrophic fungi‐based food chains. Briones M, editor. Funct Ecol. 2020;34: 2375–2388. doi:10.1111/1365-2435.13654

25. Pollierer MM, Larsen T, Potapov A, Brückner A, Heethoff M, Dyckmans J, et al. Compound-specific isotope analysis of amino acids as a new tool to uncover trophic chains in soil food webs. Ecol Monogr. 2019;89: e01384. doi:10.1002/ECM.1384

26. Scott JH, O’Brien DM, Emerson D, Sun H, McDonald GD, Salgado A, et al. An examination of the carbon isotope effects associated with amino acid biosynthesis. Astrobiology. 2006;6: 867–880. doi:10.1089/AST.2006.6.867

27. Thorp JH, Bowes RE. Carbon Sources in Riverine Food Webs: New Evidence from Amino Acid Isotope Techniques. Ecosystems. 2017;20: 1029–1041. doi:10.1007/s10021-016-0091-y

28. Vane K, Cobain MRD, Trueman CN, Vonnahme TR, Rokitta S, Polunin NVC, et al. Tracing basal resource use across sea-ice, pelagic, and benthic habitats in the early Arctic spring food web with essential amino acid carbon isotopes. Limnol Oceanogr. 2023;68: 862–877. doi:10.1002/LNO.12315
